# Combining ERBB family and MET inhibitors is an effective therapeutic strategy in cutaneous malignant melanoma independent of *BRAF/NRAS* mutation status

**DOI:** 10.1038/s41419-019-1875-8

**Published:** 2019-09-10

**Authors:** Ishani Das, Margareta Wilhelm, Veronica Höiom, Rodolfo Franco Marquez, Fernanda Costa Svedman, Johan Hansson, Rainer Tuominen, Suzanne Egyhàzi Brage

**Affiliations:** 1 Department of Oncology and Pathology, Karolinska Institutet, Stockholm, Sweden; 2 Department of Microbiology, Tumor and Cell Biology, Karolinska Institutet, Stockholm, Sweden; 3 Department of Pathology and Cytopathology, Hospitatal Univeristario, UANL, Nuevo Leon, Mexico; 4 Karolinska University Hospital, Stockholm, Sweden

**Keywords:** Targeted therapies, Melanoma

## Abstract

Current treatment modalities for disseminated cutaneous malignant melanoma (CMM) improve survival; however, relapses are common. A number of receptor tyrosine kinases (RTKs) including EGFR and MET have been reported to be involved in CMM metastasis and in the development of resistance to therapy, targeting the mitogen-activated protein kinase (MAPK pathway). IHC analysis showed that patients with higher MET protein expression had a significantly shorter overall survival. In addition, silencing of MET caused an upregulation of EGFR and p-AKT, which was abrogated by concomitant silencing of MET and EGFR in CMM cells resistant to MAPK-targeting drugs. We therefore explored novel treatment strategies using clinically approved drugs afatinib (ERBB family inhibitor) and crizotinib (MET inhibitor), to simultaneously block MET and ERBB family RTKs. The effects of the combination were assessed in cell culture and spheroid models using established CMM and patient-derived short-term cell lines, and an in vivo xenograft mouse model. The combination had a synergistic effect, promoting cell death, concomitant with a potent downregulation of migratory and invasive capacity independent of their *BRAF/NRAS* mutational status. Furthermore, the combination attenuated tumor growth rate, as ascertained by the significant reduction of Ki67 expression and induced DNA damage in vivo. Importantly, this combination therapy had minimal therapy-related toxicity in mice. Lastly, the cell cycle G2 checkpoint kinase WEE1 and the RTK IGF1R, non-canonical targets, were altered upon exposure to the combination. Knockdown of WEE1 abrogated the combination-mediated effects on cell migration and proliferation in *BRAF* mutant BRAF inhibitor-sensitive cells, whereas WEE1 silencing alone inhibited cell migration in *NRAS* mutant cells. In summary, our results show that afatinib and crizotinib in combination is a promising alternative targeted therapy option for CMM patients, irrespective of *BRAF/NRAS* mutational status, as well as for cases where resistance has developed towards *BRAF* inhibitors.

## Introduction

After the discovery of *BRAF*-activating mutations in around 50% of cutaneous malignant melanoma (CMM) patients^1,2^, the development of therapies for disseminated *BRAF*-mutated CMM has shown promising clinical results. Although therapy combining a BRAF inhibitor with a MEK inhibitor increases the median Progression Free Survival (PFS) to ~11 months and results in long-term survival in around 30% of the patients^3^, relapses are still common. Approximately 30% of CMM patients present tumors with *NRAS* mutations^4,5^ and are therefore not eligible for inhibitors of mutated BRAF, as these drugs appear to be tumor promoting for these patients^6^, necessitating alternate therapy approaches for targeted therapy. Immunotherapy with checkpoint inhibitors has been successful for a subset of CMM patients. Although treatment with checkpoint inhibitors had similar effect on patients with *NRAS* mutant CMM and *NRAS* wild-type (WT) CMM, median overall survival (OS) was significantly shorter for patients with NRAS mutant CMM^7^. Moreover, patients who are negative for BRAF mutations in V600 position and develop acquired therapy resistance towards immunotherapy are left with few good alternatives for treatment^8^.

Previous studies have shown that some of the mechanisms by which CMM with *BRAF* V600 mutations become drug resistant against BRAF or MEK inhibitors involve upregulation of receptor tyrosine kinases (RTKs) such as MET^9^ and epidermal growth factor receptor (EGFR)^4^. It has also previously been demonstrated that MET could be a mechanism of resistance to EGFR inhibitor, which could be mediated by a crosstalk between MET and EGFR^10^. The presence of an EGFR-T790M mutation in lung cancer can also lead to the development of EGFR inhibitor resistance but afatinib, targeting ERBB family receptors, can overcome this specific EGFR inhibitor resistance. However, in cells with MET amplification, this resistance can be overcome by combining afatinib with the MET/ALK inhibitor crizotinib^11^.

In this study we aimed to investigate whether afatinib together with crizotinib could be a potential novel combination treatment for BRAF inhibitor-sensitive and -resistant CMM, as well as for *NRAS* mutant and *BRAF/NRAS* WT CMM. To explore the therapeutic potential of this novel drug combination, we performed different functional assays to determine the combination effects on cell death, invasion, migration, and proliferation. To ascertain whether differences in molecular signaling patterns could explain the varied combination treatment responses observed between cell culture and spheroid models, western blotting was conducted. To elucidate the in vivo relevance of our study, we employed a xenograft animal model. Lastly, a network analysis followed by protein expression analysis was performed to reveal novel potential drug targets.

## Results

### MET and ERBB3 is highly expressed in metastatic CMM

**Fig. 1:**
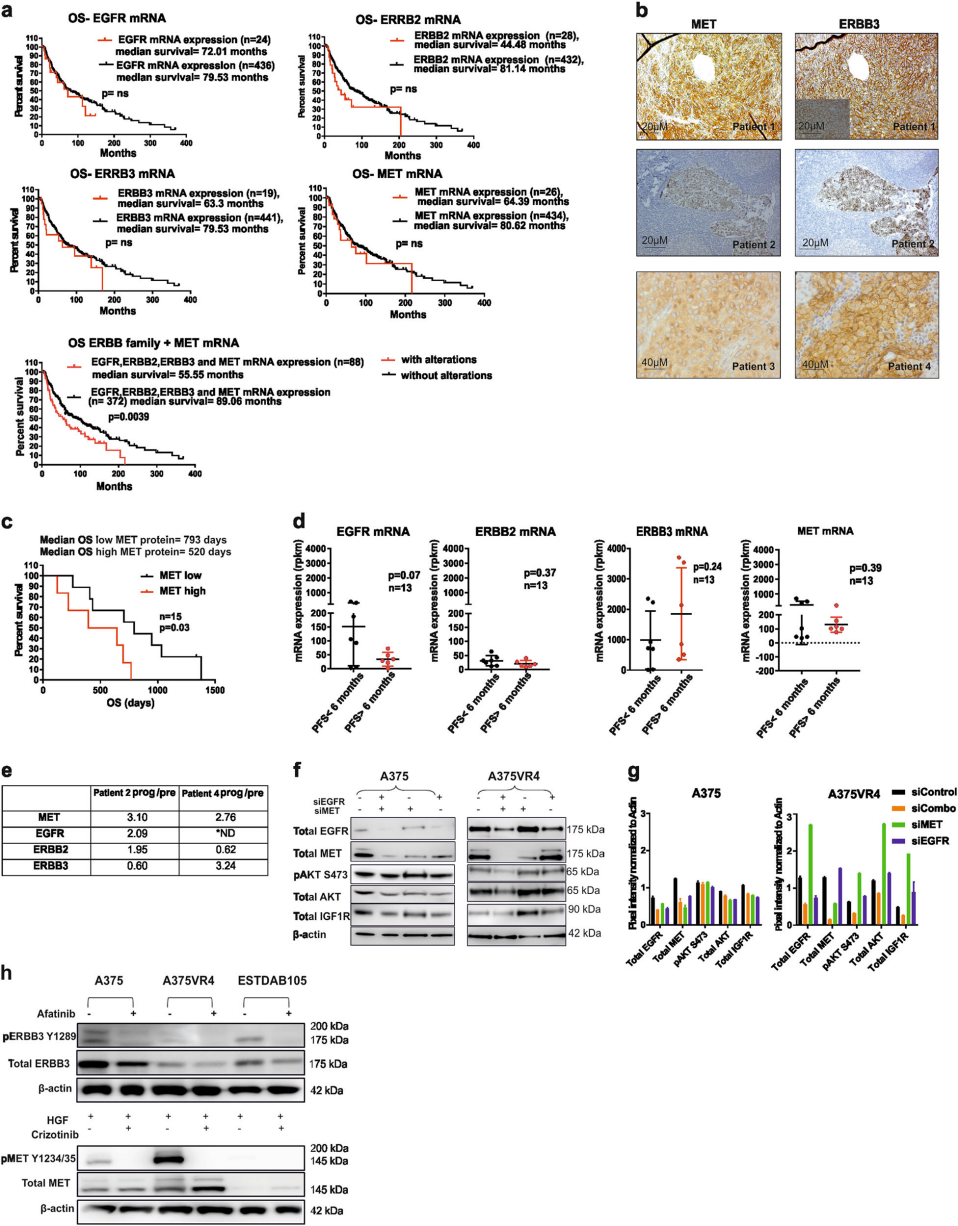
Protein and mRNA expression of MET and ERBB family RTKs in CMM. **a** Kaplan–Meier analysis of TCGA dataset relating changes in mRNA of MET and ERBB family members to OS in CMM alone or together (*n* = 461). **b** Images showing membranous and cytoplasmic protein expression of MET and ERBB3 in CMM. **c** Kaplan–Meier analysis showing effects of high or low MET protein expression on OS in CMM patients (*n* = 15) (Log-rank (Mantel–Cox) test). **d** AmpliSEQ data comparing PFS (<6 months or >6 months) after MAPK targeting treatment with relative mRNA expression of known afatinib and crizotinib targets in pre-treatment clinical samples (*n* = 13). **e** Relative mRNA expression in MET and ERBB family RTKs in matched metastases taken before the start of treatment with vemurafenib and after progression from two CMM patients. **f** Western blotting showing that knockdown of MET leads to the upregulation of EGFR, IGF1R, pAKT, and total AKT expression in vemurafenib-resistant subline A375VR4, which is blocked by co-silencing MET and EGFR. **g** Quantification of Fig. 1f using ImageJ. **h** Western blottings showing that afatinib targets p-ERBB3 and crizotinib targets pMET. *ND = Not detected. All samples are expressed as mean ± SD. All experiments were repeated in triplicates. *p < 0.05, **p < 0.01, ***p < 0.001, ****p < 0.0001 as determined by two-tailed Student’s *t*-test

Upregulation of RTK EGFR, MET, and ERBB3 have previously been reported to be involved in CMM metastasis and development of resistance to mitogen-activated protein kinase (MAPK)-targeted therapy^4,12–15^. The Cancer Genome Atlas Program (TCGA) analysis revealed that alteration of the ERBB (EGFR, ERBB2, and ERBB3) and MET mRNA expression together is associated with significantly shorter OS but not alone (Fig. 1a)^16,17^. Metastatic CMM tumors displayed moderate to high cytoplasmic and membranous ERBB3 and MET expression in 12/13 (92%) and 9/21 (43%) *BRAF*-mutated tumor samples, respectively (Fig. 1b, Supplementary Table 1). Overall, EGFR signal was relatively weak in our sample set and few of the samples had high cytoplasmic and membranous staining (3/21; 14%); however, in an additional number of tumors scattered, high expressing tumor cells were observed (Supplementary Fig. S1, Supplementary Table 1). Survival analysis confirmed that CMM patients with high MET expression in pre-treatment tumor biopsies have concomitantly shorter OS (*n* = 15, *p* = 0.026) (Fig. 1c). We performed transcriptional profiling of clinical *BRAF*-mutated pre-treatment samples (*n* = 13) (MAPK-targeting drugs), which showed a nonsignificant tendency towards higher mRNA expression of EGFR, MET, and ERBB2, which was associated with shorter PFS, whereas an opposite tendency was found for ERBB3 (Fig. 1d, Supplementary Table 2a). Furthermore, transcriptional profiling of sequentially sampled *BRAF*-mutated tumors (identical body location in sampling) (*n* = 2) from CMM patients treated with vemurafenib showed induction of MET and EGFR/ERBB3 mRNA at relapse (Fig. 1e)^18^.

### Afatinib and crizotinib combination regime attenuates cell viability in CMM cells

To investigate whether dual inhibition of ERBB family members and MET could prevent induction of compensatory pathways, we first silenced MET or EGFR in BRAF mutant cells to see whether silencing of one RTK affected the expression of the other. Here we observed that knockdown of MET caused an upregulation of EGFR in A375VR4, validating a previously shown crosstalk between MET and EGFR^12^. EGFR upregulation also had downstream effects as indicated by an induction of p-AKT levels. Interestingly, we also saw that simultaneous knockdown of EGFR and MET did not cause an upregulation of pAKT or total AKT (Fig. 1f, g) supporting the rationale for using MET and EGFR inhibitors in combination. As our pre-treatment CMM samples had a high protein expression of ERBB3 (Fig. 1b, Supplementary Table 1) and EGFR, ERBB2 was upregulated in patient 2 at relapse, whereas ERBB3 was upregulated in patient 4 at relapse (Fig. 1e); thus, we decided to use a pan-ERBB inhibitor. Previous studies have shown that afatinib also inhibits ERBB3^19^. Immunoblotting performed on selected cell lines confirmed p-ERBB3 as a target for afatinib and p-MET as a target for crizotinib (Fig. 1h). Short-term exposure with crizotinib resulted in a decrease in pAKT and total AKT, although no effect on pERK or total ERK was observed (Supplementary Fig. S2).

**Fig. 2:**
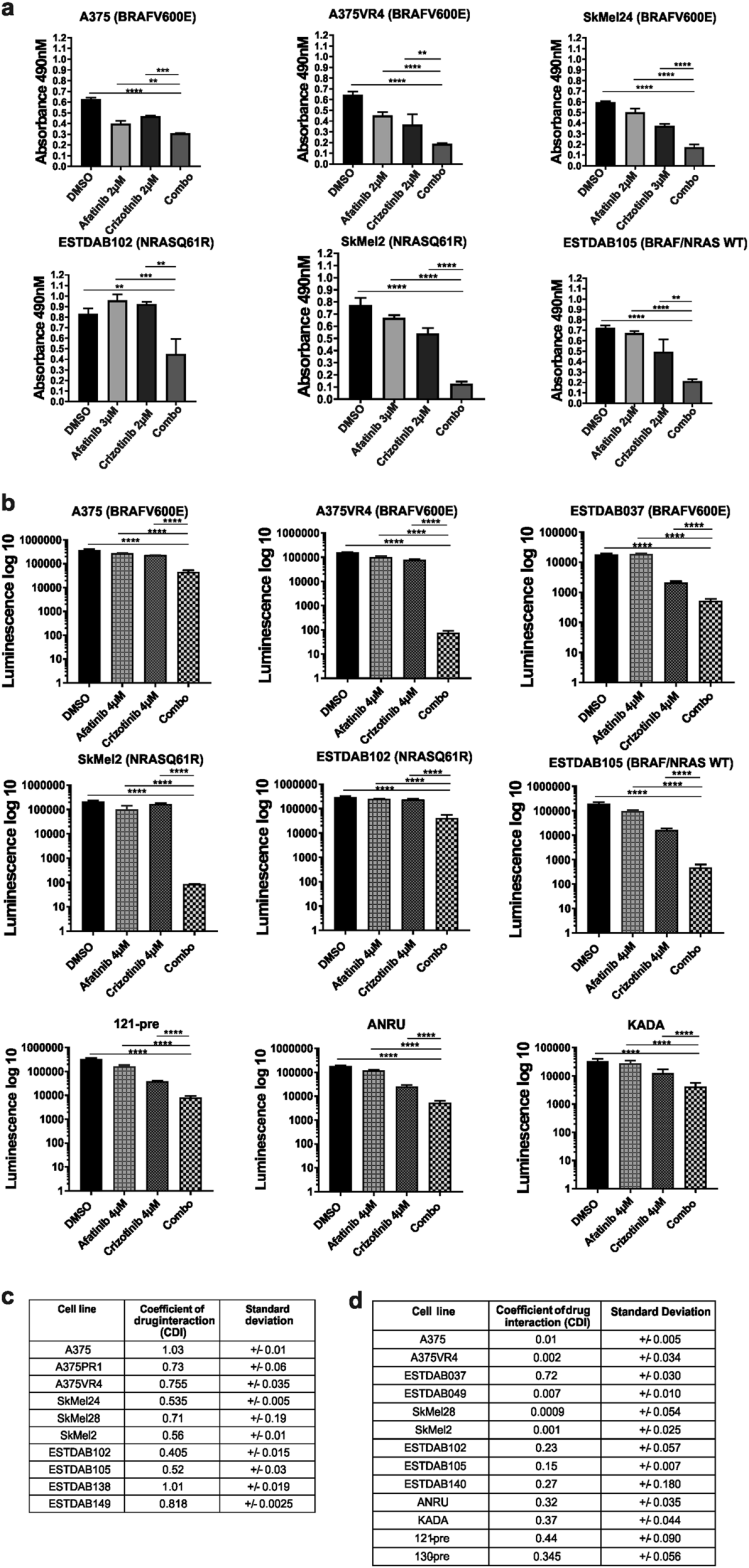
Combination of afatinib and crizotinib in 2D and 3D cell viability assays. **a** Effects of combination therapy on CMM cells was measured by 2D viability assay. **b** Effects of combination therapy on 3D spheroids was measured by 3D viability assay. **c**, **d** Tables are showing CDI of the combination therapy for 2D and 3D models, respectively. CDI < 1 = synergy, CDI = 1 = additive effect, CDI > 1 = antagonism. All samples are expressed as mean ± SD. All experiments were repeated in triplicates. *p < 0.05, **p < 0.01, ***p < 0.001, ****p < 0.0001 as determined by two-tailed Student’s *t*-test

We employed the use of afatinib and crizotinib to study the effects of the drugs alone or in combination on a panel of CMM cell lines. In vitro inhibitory concentration at 50% for afatinib or crizotinib alone was first calculated in ten CMM cell lines with variable *BRAF* and *NRAS* mutation status, including cell lines with intrinsic or acquired BRAF inhibitor resistance. The IC30 concentrations were used for most of the combination analyses (Supplementary Table 3). Drug synergy assay conducted on four CMM cell lines showed an overall synergistic score (Supplementary Fig. S3), which remained true for three of the four cell lines when calculating coefficient of drug interaction (CDI). In five of six additional CMM cell lines, a synergistic effect was also observed (Fig. 2a, c, Supplementary Fig. S5a). To further validate this observation, a three-dimensional (3D) model of tumor cell spheres was employed (Supplementary Fig. S4). After dose optimization, spheres from 13 different CMM cell lines treated with either afatinib or crizotinib alone or in combination showed similar cytotoxic effects as observed in two dimension (2D) (Fig. 2b, Supplementary Fig. S5b) with CDI values confirming synergism for the tested concentrations for all CMM cell lines (Fig. 2d).

### Afatinib and crizotinib combination treatment decreases cell proliferation and induces apoptosis in CMM cells

**Fig. 3:**
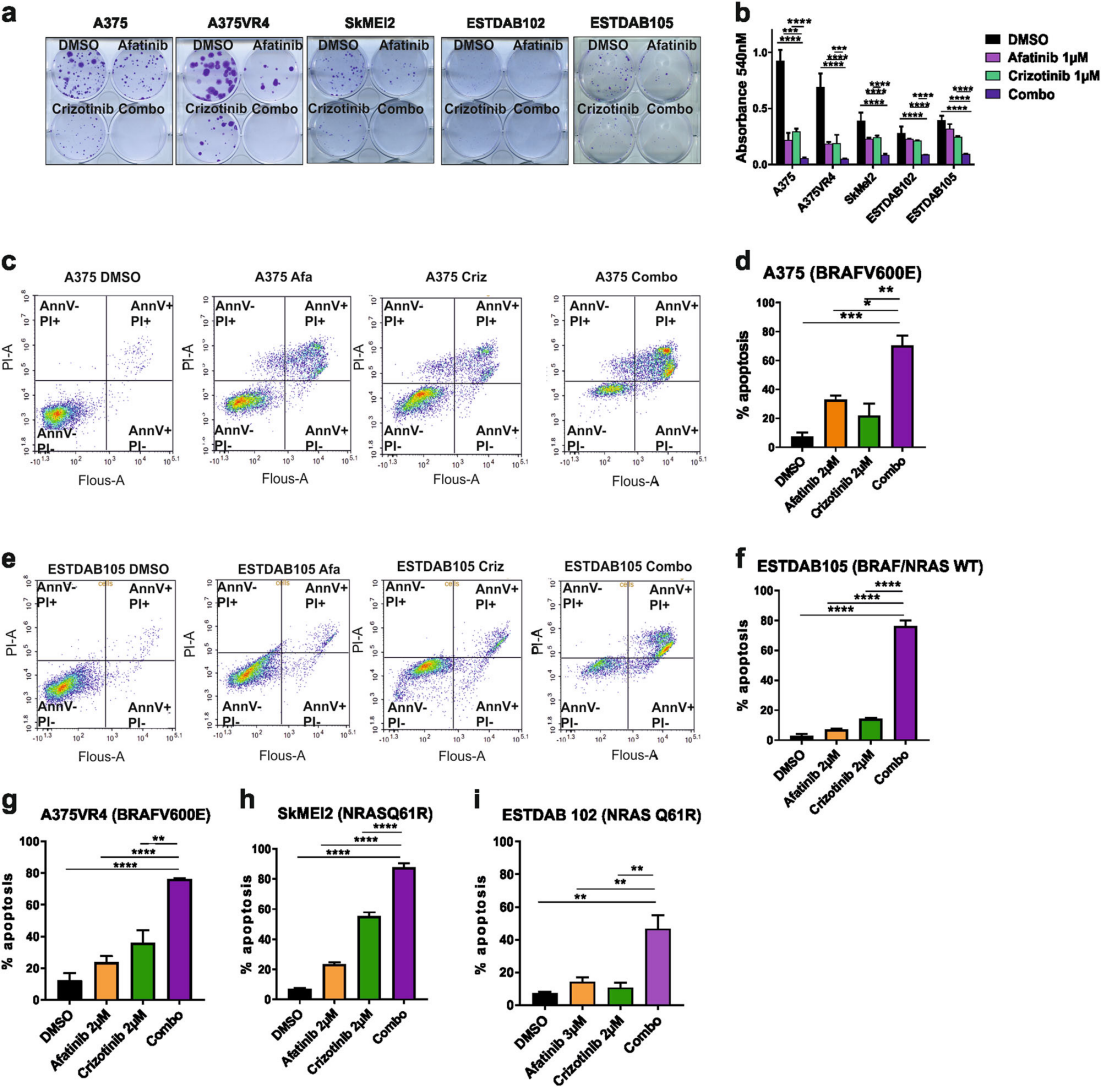
Combination treatment induces apoptosis in CMM cells. **a** Colony formation in a 2D model system is either inhibited or totally abolished in CMM cells irrespective of *BRAF/NRAS* mutation status after combination treatment. **b** Quantification of colony formation assay after single or combination treatment. **c** Induction of apoptosis as measured by FACS using Annexin V and PI in A375 after single or combination treatment for 72 h (representative image from one experiment). **d** Quantification of percentage of apoptotic cells. **e** Induction of apoptosis as measured by FACS using Annexin V and PI in ESTDAB105 after single or combination treatment for 72 h (representative image from one experiment). **f** Quantification of percentage of apoptotic cells using an average of three independent experiments. **g**–**i** Quantification of percentage of apoptotic cells using an average of three independent experiments for A375VR4, SkMel2 and ESTDAB102. All samples are expressed as mean ± SD. All experiments were repeated in triplicates. *p < 0.05, **p < 0.01, ***p < 0.001, ****p < 0.0001 as determined by two-tailed Student’s *t*-test

Both the ERBB family kinases and MET have previously been implicated in CMM growth and proliferation^20,21^. In order to further investigate the effects of the drug combination on proliferation, five cell lines were selected (A375, A375VR4, SkMel2, ESTDAB102, ESTDAB105) and treated with afatinib or crizotinib alone or in combination using colony formation assay (Fig. 3a). Single treatments were in most cases able to reduce colony formation, whereas the combination treatment almost completely abolished the formation of colonies (Fig. 3a). Quantification demonstrated that in A375 and A375VR4, the combination readout was 10% of the vehicle control absorbance (*p* < 0.0001). The combination treatment in the *BRAF* WT cell reduced the readings to <20% compared with dimethyl sulfoxide (DMSO) (*p* < 0.0001) (Fig. 3b).

To study whether the reduction in cell proliferation was due to apoptotic cell death, we performed fluorescence-activated cell sorting (FACS) analysis after 72 h treatment. A significant induction of apoptosis with the combination was observed, compared with single treatments (*p* < 0.05) (Fig. 3c–i). However, in both A375 and A375VR4, only late apoptosis was observed. This was in contrast to the remaining cell lines displaying both early and late apoptosis.

**Fig. 4:**
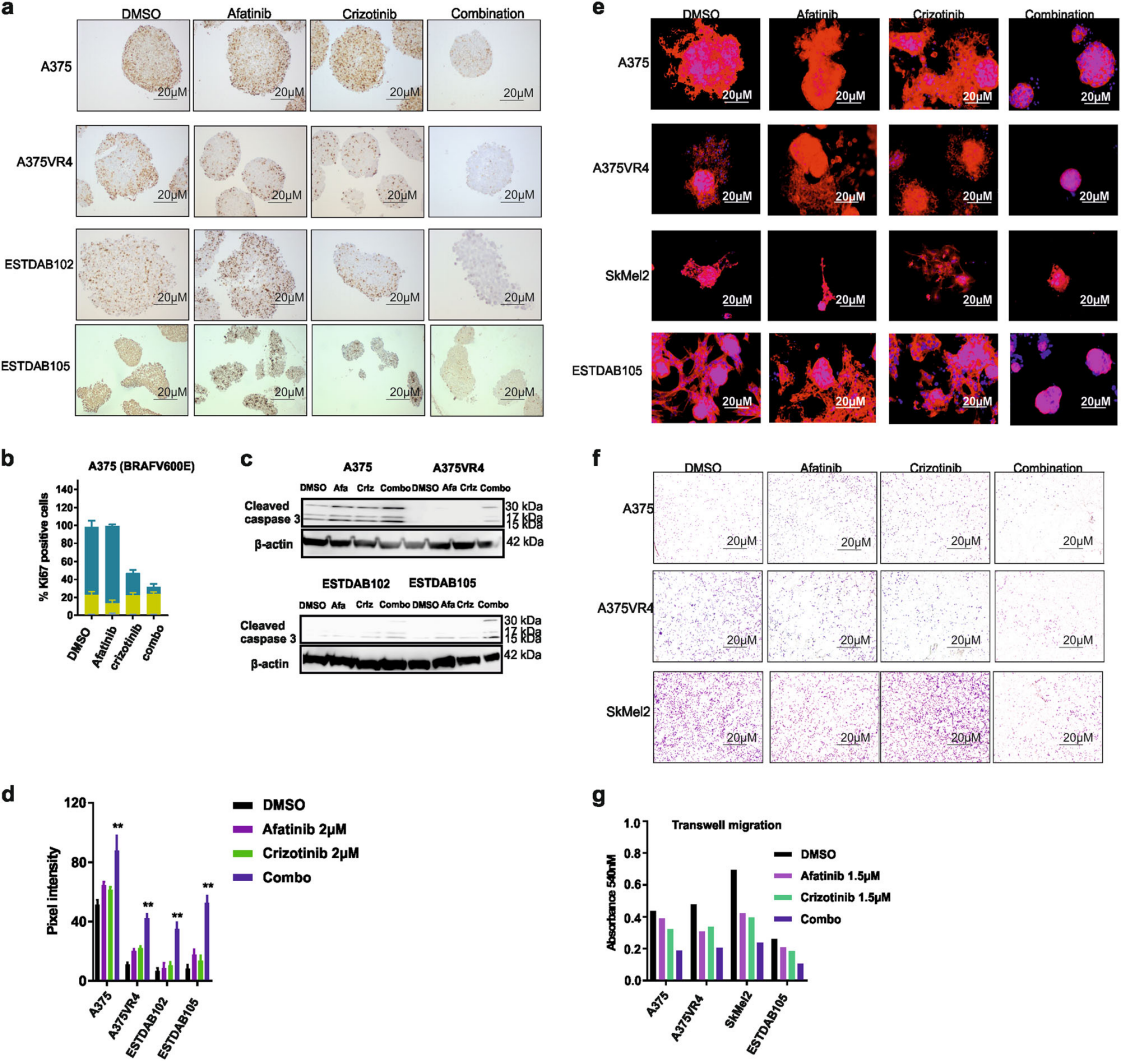
Combination of afatinib and crizotinib reduces colony formation, proliferation, invasion, and induces caspase activity in vitro. **a** ICC showing loss of proliferation marker Ki67 in 3D spheres for A375, A375VR4, ESTDAB102, and ESTDAB105 cell lines. **b** Overall score for all spheres on each slide as determined by ICC. **c** Western blotting showing induction of cleaved caspase 3 in 3D spheres after treatment for 72 h with either afatinib or crizotinib 2 µM single treatment or combination. **d** Quantification of **c** using ImageJ software. **e** Invasion in a 3D spheroid model of CMM cells are inhibited when treated with afatinib and crizotinib at 2 µM of each drug in combination. **f** Transwell migration assay showing the combination treatment was most efficient in reducing migratory capacity in CMM cells. **g** Quantification of **f**. The ICC was repeated in duplicate and the invasion assay was repeated in triplicate

A spheroid model was employed to ascertain the observed drug effects on proliferation and cell death. Treatment with either crizotinib alone or the combination caused an overall decrease in Ki67 signal, whereas afatinib alone did not (Fig. 4a). A more potent decrease was observed after combination treatment compared with crizotinib alone in A375, A375VR4, and ESTDAB102. In ESTDAB105, the individual drugs caused a similar loss in proliferation index as the combination treatment (Fig. 4b). In addition, p-H2AX induction was observed in three of four cell lines compared with the levels in control or single drug exposures (Supplementary Fig. S6). Western blotting confirmed that the combination led to induction of cleaved caspase 3 across all cell lines (Fig. 4c, d), which further corroborated our FACS results (Fig. 3c–i).

### Single and combination treatments lead to the formation of autophagosomes

Autophagy can have a cytoprotective role in the tumor cells, which can be enhanced by MET and EGFR inhibitors^22,23^. To validate whether afatinib and crizotinib also induced autophagy in CMM cells and investigate whether there may be a difference between single and combination treatment, we performed transmission electron microscopy. Our results demonstrated that afatinib or crizotinib alone, or the combination, increased autophagosome numbers compared with DMSO. However, no apparent increase in autophagosome numbers for single vs. combination treatments could be ascertained and was therefore not studied further (Supplementary Fig. S7).

### Combination treatment with afatinib and crizotinib reduces invasive and migratory capacity of CMM cells

Dual inhibition of EGFR and MET has previously been demonstrated to suppress invasion of cancer cells^10^. We observed that CMM cells cultured in 3D displayed a complete abrogation of invasion after combination treatment when compared with DMSO or single treatments (Fig. 4e). For ESTDAB105, single drugs did not have any pronounced effect on the spheroid invasive capacity. Surprisingly, treatment with crizotinib in SkMel2 caused only a partial loss in invasiveness, despite this cell line being highly sensitive to crizotinib.

Increased expression levels of MET, EGFR, and ERBB3 have been previously associated with increase in both CMM progression and metastasis^24,25^. To study the effects mediated by drug combination on cell migration, we performed a scratch assay. All cell lines displayed significantly reduced cell migration capacity, here seen by the time required to close the wound gap (*p* < 0.01) (Supplementary Fig. S8). To confirm these results, we also performed a transwell migration assay. Overall, the combination treatment was able to reduce the migratory capacity of all cell lines tested (Fig. 4f, g).

### Combination treatment with afatinib and crizotinib abrogates tumor growth rate in vivo

**Fig. 5:**
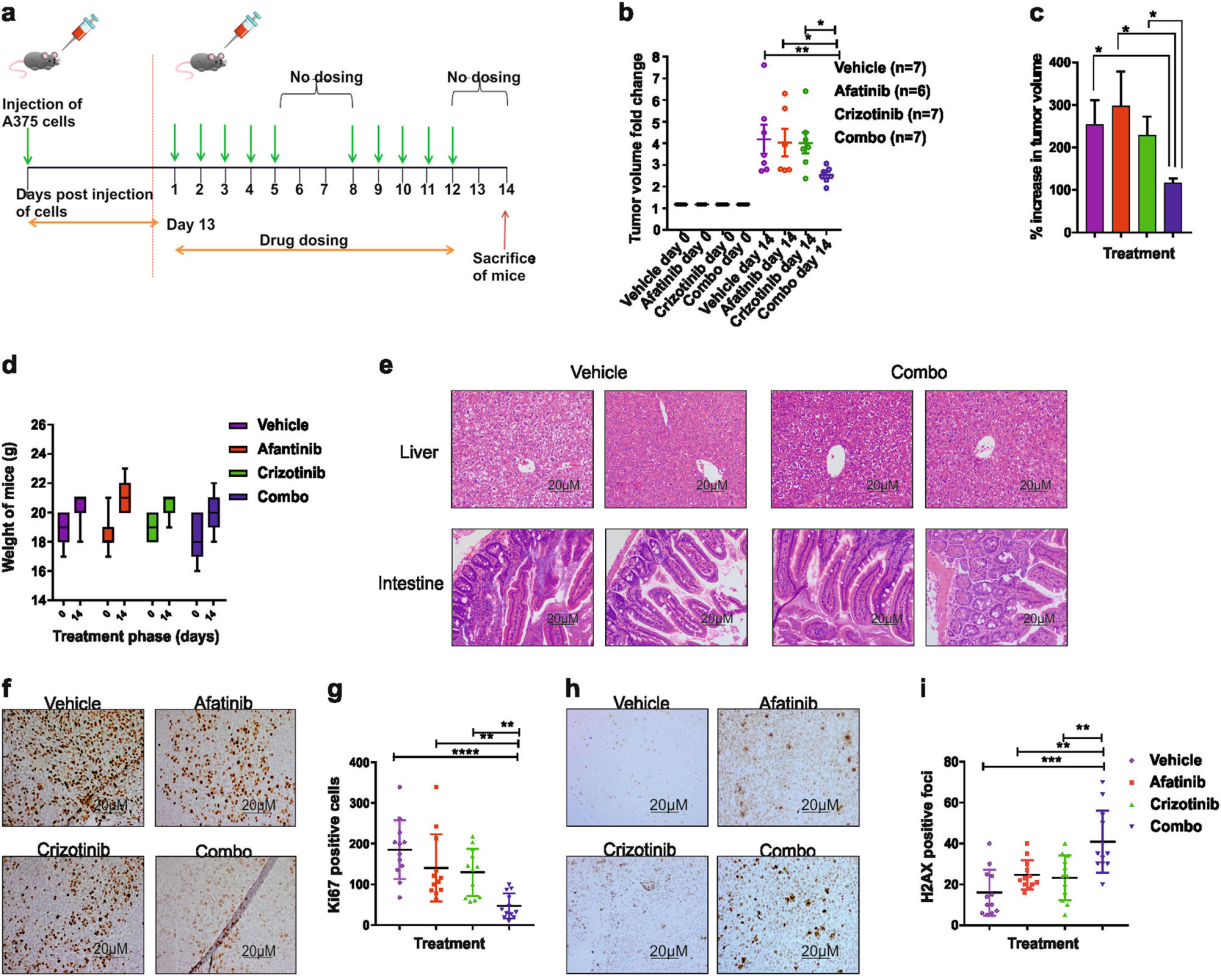
Combination treatment was the most effective in decreasing tumor growth rate in vivo. **a** Schematic of the dosing schedule followed. Afatinib was used at 20 mg/kg, crizotinib at 15 mg/kg, and the combination treatment was afatinib and crizotinib (20 mg/kg + 15 mg/kg). **b** Tumor volume fold change day 0 vs. day 14. **c** Increase in percentage (%) of tumor volume calculated as the tumor volume at the end of treatment (day 14) compared with the start of treatment (day 0). **d** Weight of animals at the start and end of treatment. **e** HTX pictures comparing liver and intestine in vehicle-treated vs. combination-treated mice. **f** IHC showing Ki67. **g** Quantification of Ki67-positive cells. **h** IHC showing p-H2AX. **i** Quantification of p-H2AX. All samples are expressed as mean ± SEM. For IHC quantification, samples are expressed as mean ± SD. Differences between animal groups were calculated by the Mann–Whitney test. For IHC staining (*n* = 3), differences were calculated by two-tailed Student’s *t*-test *p < 0.05, **p < 0.01, ***p < 0.001, ****p < 0.0001

To validate the efficacy of the combination treatment from our in vitro data, a xenograft mouse model was employed using A375 cells (Fig. 5a). In contrast to our in vitro FACS experiments where afatinib or crizotinib individually caused 40% and 25% cell death, respectively, we saw no decrease of the tumor volume with the single treatments when compared with vehicle control in our xenograft model. However, the tumor volumes in the combination treatment group increased significantly slower (<50% compared with any of the treatment arms) when compared with the other treatment arms and vehicle control (*p* < 0.05) (Fig. 5b, c, Supplementary Fig. 9a). The treatment did not have any significant impact on the tumor weight (Supplementary Fig. 9c-e), which may be attributed to the fact that the median tumor volume of the combination treatment arm at the start of treatment was 31.25% more than that of the vehicle and crizotinib arms, and 8.75% more than that of the afatinib arm (Supplementary Fig. 9b). As the combination treatment used has shown to be toxic to animals in terms of weight loss and damage to the intestine in a previously published lung cancer study^11^, we also investigated for the same in our animal model, although we have chosen to use lower concentrations of afatinib (20 mg/kg) and crizotinib (15 mg/kg) than in this previous report (25 mg/kg afatinib + 25 mg/kg crizotinib). Our study design did not cause any toxicity to animals in terms of weight (Fig. 5d) and the histopathology of the liver and intestine were unaltered (Fig. 5e). The effectiveness of the combination treatment was further manifested by the significant decrease in the proliferation index (*p* < 0.01) (Fig. 5f, g) and the significant increase in DNA damage marker p-H2AX (*p* < 0.01) (Fig. 5h, i) when compared with either single treatments or the vehicle control. This is in line with our in vitro findings using the spheroid model (Fig. 4a, Supplementary Fig. S6).

### WEE1 and IGFR show pronounced reduced expression after combination treatment

**Fig. 6:**
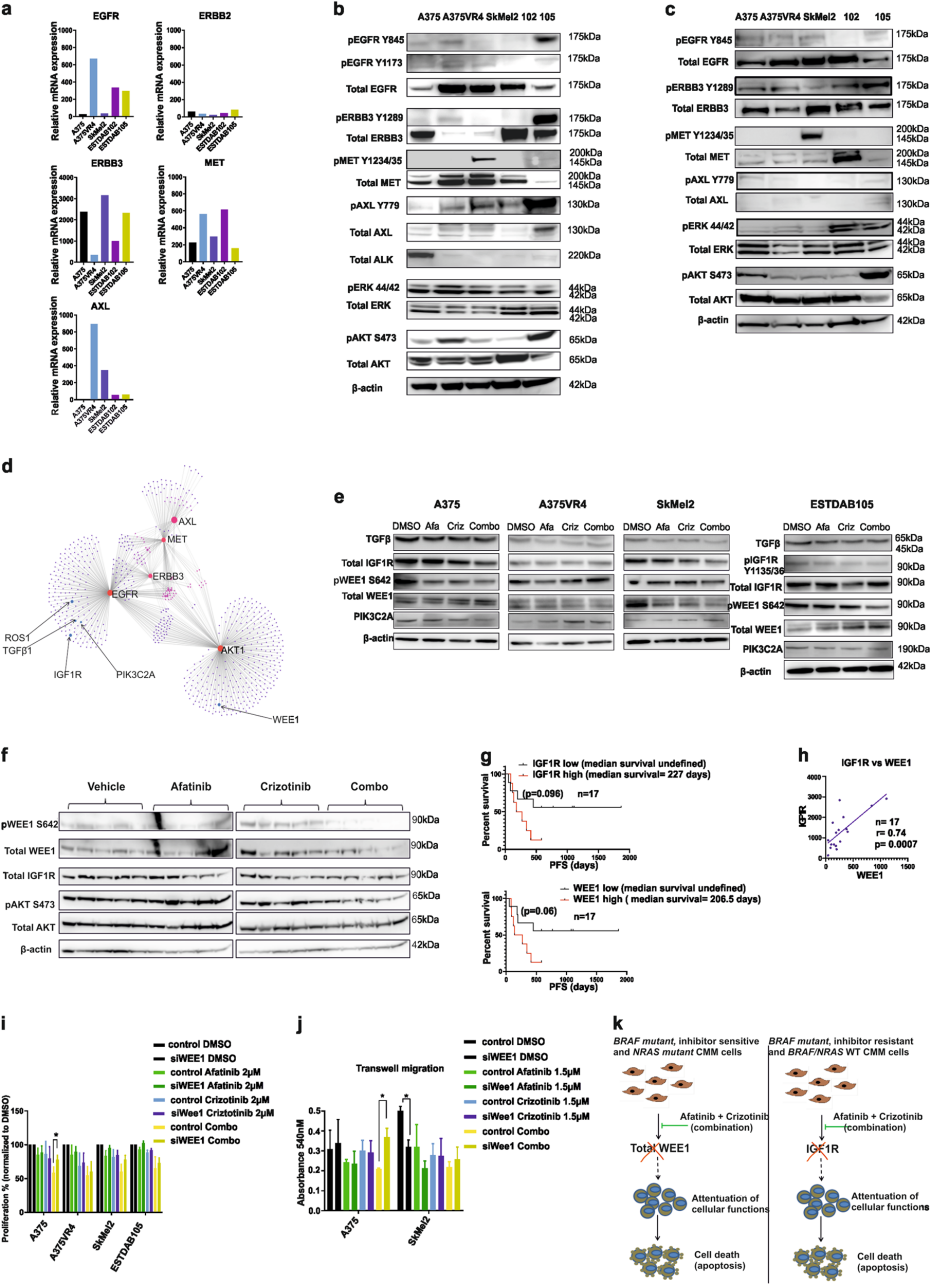
Drug combination effects are not limited to the canonical targets. **a** AmpliSEQ data showing relative mRNA expression of known afatinib and crizotinib targets in five CMM cell lines. **b** Western blotting showing total expression and phosphorylation status of known targets of afatinib and crizotinib, and total expression and phosphorylation status of ERK and AKT proteins after 2D culturing. **c** Western blotting showing total expression and phosphorylation status of known targets of afatinib and crizotinib, and phosphorylation status of ERK and AKT proteins after 3D culturing. **d** Pathway analysis showing possible secondary hits of the drugs. **e** Western blottings showing changes in expression patterns of these secondary hits upon exposure to drugs. **f** Western blotting using xenograft samples (*n* = 20) showing reduction of WEE1 in the combination treatment arm. **g** AmpliSEQ analysis of pre-treatment tumors from CMM patients (*n* = 17) who received checkpoint inhibitors show association of high mRNA expression levels of IGF1R and WEE1 with a shorter OS, although not statistically significant. **h** IGF1R and WEE1 mRNA expression is significantly correlated in these CMM (Fig. 6g). **i** Knockdown of WEE1 abrogates the reduction in proliferation mediated by combination treatment in both A375 and SkMel2. **j** Silencing WEE1 also rescues the migratory capacity of A375 cells. **k** Schematic of the drug combination effects in CMM. All in vitro experiments were repeated in triplicates

Validation of the mutational background of cell lines used in this study was done using whole genome sequencing (WGS) and data are shown here using the mutation mapper^16,17^ for four out of the five cell lines more extensively investigated in the study (Supplementary Fig. S10, Supplementary Table 4). Baseline mRNA expression of the drug targets and key downstream players indicated that BRAF inhibitor-induced resistance caused an upregulation of *EGFR* (25-fold) and *MET* (2.1-fold), and downregulation of ERBB3 (8-fold) in A375VR4 cells, compared with its vemurafenib-sensitive A375 (Fig. 6a, Supplementary Table 2b-c). In *NRAS* mutant lines, high levels of *EGFR* and *MET* were observed in ESTDAB102, whereas SkMel2 had high levels of *ERBB3*. WT cell line ESTDAB105 displayed high levels of *AXL*, *EGFR*, and *ERBB3*. Western blot analysis confirmed high protein levels of EGFR, pERBB2, and MET in A375VR4 compared with that in A375 (Fig. 6b, Supplementary Fig. S11). High MET expression was also observed in SkMEl2, whereas the remaining two lines had high EGFR and ERBB3. The protein expression levels were also validated in 3D spheroids (Fig. 6c). Differences in expression patterns were observed when compared with the 2D model. Strikingly, the levels of MET expression was lower (except for ESTDAB102 and ESTDAB105), whereas a marked reduction in pAKT signal was observed in A375VR4 spheroids.

To elucidate the role of crizotinib (as a MET inhibitor), we silenced MET (Supplementary Table 4). However, silencing of MET could not further sensitize *BRAFV600E* mutant cell lines towards afatinib (Supplementary Fig. S12a-b), indicating that the drugs might have novel non-canonical targets. Network analysis performed on canonical targets of afatinib and crizotinib (Fig. 6d) revealed potential candidates, which have been previously associated with CMM. To investigate this, we conducted a short drug exposure (3 h) ensued by western blot analysis, to capture relatively direct effects of the drugs (Fig. 6e). WEE1, which has been previously associated with G2/M cell cycle arrest in CMM^26^, showed the most pronounced reduction of phosphorylated WEE1 signal after a combination treatment in A375 and SkMel2. Moreover, a similar downregulation of the total WEE1 was seen in ESTDAB105. TGFβ has been identified as an attractive therapeutic candidate in CMM because of its role in CMM progression^27^. A decrease in the expression of TGFβ protein was observed in SkMel2 and ESTDAB105 after treatment. The strongest reduction of total RTK IGF1R was observed in A375 and ESTDAB105 after the combination treatment, whereas in SkMel2, only crizotinib caused a reduction of IGF1R expression. The impact of PI3K/AKT pathway in CMM has been investigated in a recent research publications^28,29^. Our analysis showed a reduced signal of PI3KC2a upon combination treatment, only in A375. To test whether WEE1 and IGF1R were reduced upon combination treatment in vivo, we performed a western blot analysis. We observed that the combination treatment was able to downregulate pWEE1 and total WEE1 in our A375 xenograft model. IGF1R signal was overall reduced in the combination treatment arm in the xenograft model, although there was no striking difference between the crizotinib treatment arm vs. the combination treatment arm (Fig. 6f). Furthermore co-silencing of MET and EGFR reduced the IGF1R expression in vitro in A375VR4, while silencing MET or EGFR alone induced IGF1R expression (Fig. 1f, g). Recently, Sun et al.^30^ demonstrated that inhibition of WEE1 sensitized different cancer models to checkpoint inhibitors. Results from a small set of pre-treament CMM samples from patients who received checkpoint inhibitors support the finding of Sun et al.^30^, wherein high WEE1 mRNA was associated with shorter OS, although not reaching statistical significance (*p* = 0.06). IGF1R mRNA expression showed a similar association to OS and was significantly correlated to WEE1 mRNA expression (*p* = 0.007) in these CMM samples (*n* = 17) (Fig. 6g, h).

### Downregulation of WEE1 reduces combination-mediated effects on proliferation and migration in *BRAF*-mutated CMM cells and migration in *NRAS*-mutated cells

As WEE1 has been previously associated with many cancer types including CMM^31,32^, we wanted to further investigate the functional role of WEE1. We silenced WEE1 and checked for its potential role in mediating combination-related effects on proliferation and migration. Indeed, silencing of WEE1 reduced pAKT signaling and induced pH2AX signal in all cell lines (Supplementary Fig. S13). Knocking down WEE1 significantly diminished the combination-mediated effects on proliferation and migration in A375. Combination treatment-mediated effects on proliferation were also reduced in SKMEl2, although not significant (*p* = 0.09) (Fig. 6i). Silencing of WEE1 alone in SkMEl2 reduced migratory capacity (Fig. 6j).

## Discussion

The present study is, to our knowledge, the first example demonstrating an effect of the United States Food and Drug Administration (FDA)/European Medicines Agency (EMA)-approved lung cancer drugs afatinib and crizotinib in combination on CMM cells. The observed highly synergistic reduction of viability of the CMM cells after combination exposure, regardless of their BRAF/NRAS oncogenic mutational profile, compared with single-agent exposure was to a large extent associated with impaired proliferation and increased apoptotic death. The successful reduction of tumor growth rate by the combination was also observed in a xenograft mouse model, which indicates the combined treatment to be more promising from a clinical perspective. Moreover, we also show that WEE1 and IGF1R, previously suggested as targets to potentiate CMM therapy^33–35^, are downregulated in A375/SKMEl2 and A375/ESTDAB105, respectively, in response to the combination (Fig. 6k), and that silencing Wee1 reduces the combination-mediated effects on cell proliferation and migration.

The combined effects of the drugs can be speculated to block pathways that are utilized by CMM cells to counteract the single drug-induced cell death, here exemplified by the upregulation of EGFR when MET is silenced. A number of recent studies have investigated the potential crosstalk between the ERBB family of receptors and MET, highlighting the protein–protein interactions in the development of therapy resistance^36–38^. Clinical trials combining MET inhibitors together with irreversible ERBB inhibitors have been discussed for lung cancer and colorectal cancer^39,40^, and a recent publication has also investigated the use of MET inhibitor foretinib with EGFR inhibitor gefitinib or lapatinib in CMM cell lines^41^. Our findings support the relevance of using a combination of RTK inhibitors to target multiple RTKs, to more efficiently inhibit tumor growth and invasion of CMM.

The combination of afatinib and crizotinib in CMM cells leading to a broad phenotypic response could plausibly be related to the inhibition of multiple non-canonical targets as well. Network analysis suggested WEE1 and IGF1R to be potential candidates. Both were strongly downregulated by the combination treatment in our study. A high expression of WEE1 has been suggested to be associated with poor PFS in CMM patients^42^, thus revealing its potential role as a target for therapy^43^. In addition, the role of IGF1R-mediated therapy-induced resistance in CMM is well elucidated^15^.

The lack of severe toxicity seen with the combination of the drugs in our mouse model further supports the possible use of the combination therapy in the clinics. A previous publication on lung cancer^11^ revealed that a combination of afatinib with a high dose of crizotinib (25 mg/kg + 25 mg/kg) caused severe toxicities to the animal, both in terms of loss of weight and severe damage to the intestine. However, we did not observe any significant weight loss or liver toxicity in our animal model using afatinib (20 mg/kg) and crizotinib (15 mg/kg).This combination has previously been suggested to exceed the efficacy of any of the individual drugs for lung cancer and mesothelioma^44,45^, as corroborated in our study where treatment with afatinib together with crizotinib resulted in a significant decrease in the tumor growth rate in mice.

In conclusion, we show that the combination therapy results in highly synergistic loss of cell viability, regardless of their *BRAF/NRAS* oncogenic mutational profile, coincident with loss of invasive and migratory capacity. The reduction of tumor growth rate together with no observed toxicity in a xenograft mouse model augments the clinical applicability of the combination. Moreover, our findings suggest that the importance of WEE1 and IGF1R for the synergistic combination treatment effect should be further investigated. Future identification of key targets leading to the broad phenotypic response in the combination, in contrast to the targets of each drug as a single agent, may allow the development of novel combinatorial therapies with clinical efficacy against CMM independent of the tumor’s *BRAF/NRAS* oncogenic mutational status.

## Material and methods

### Clinical samples

Fifty-one tumor samples from 40 CMM patients, taken before the start of treatment with MAPK-targeting therapy, or after progression or before the start of treatment with checkpoint inhibitors, were collected as Formalin Fixed Paraffin Embedded (FFPE) or fresh-frozen fine-needle aspirate samples. Twenty-seven of the patients were male and 13 were female. Median age of the patients was 64 years (range 42–86 years). The CMM were classified as stage IV M1a (*n* = 3), M1b (*n* = 6), and M1c (*n* = 31). This study has obtained ethical approval from the regional ethics committee in Stockholm, Sweden, and was performed in accordance with the ethical principles given in the Helsinki Declaration. Informed consent was obtained from all the patients.

### Cell culture

Human CMM cell lines A375, SkMel24, and SkMel28 carrying the *BRAFV600E* mutation were obtained from the American Type Culture Collection; A375PR1 and A375VR4 were induced PLX4720- or vemurafenib-resistant sublines derived from A375^18^. *NRAS* mutant SkMel2 (Q61R), ESTDAB102 (Q61R), and *BRAF/NRAS* WT cell lines ESTDAB105, ESTDAB138, ESTDAB140, and ESTDAB149 were obtained from the European Searchable Tumor Line Database and Cell Bank (ESTDAB). For all experiments, CMM patient-derived cell lines 121-PRE and 130-PRE (pretreatment short-term patient-derived cell lines) originating from fine needle aspirates and CMM patient core biopsy-derived cell lines KADA and ANRU (a gift from Rolf Kiessling, Department of Oncology–Pathology, Karolinska Institutet) were cultured in Dulbecco’s modified Eagle’s medium (DMEM) supplemented with 10% fetal bovine serum (FBS), 1% sodium pyruvate, 1% non-essential amino acids, and 1% penicillin–streptomycin (Pe-St). BRAF mutant cell lines were cultured in Minimal Essential Medium supplemented with 10% FBS, 1% sodium pyruvate, 1% non-essential amino acids, and 1% Pe-St, whereas the *NRAS* and *BRAF/NRAS* WT cell lines were cultured in RPMI supplemented with 10% FBS and 1% Pe-St. For 3D MTS (3-(4,5-dimethylthiazol-2-yl)-5-(3-carboxymethoxyphenyl)-2-(4-sulfophenyl)-2H-tetrazolium) and 3D immunohistochemistry (IHC) analysis, all cells were cultured in DMEM with the same supplements as above, but were subjected to vacuum filtering with a 0.2 µm vacuum manifold filter (TPP, Switzerland). All cell lines were confirmed to be mycoplasma-free using LookOut Mycoplasma PCR detection kit (Sigma-Aldrich, Stockholm, Sweden) and the cell line authentification was performed using microsatellite fingerprinting (Eurofins Genomics, Germany).

### Xenograft model

A375 melanoma cells (3.6 × 10^6^) were mixed 1:1 with growth factor-reduced Matrigel matrix (VWR) and were injected subcutaneously in the flank of 6-week-old CB-17/Icr-*Prkdc*^*scid/scid*^ females (Janvier). Treatment started when tumors reached palpable size. Tumor size was measured three times a week using calipers; tumor volume was calculated using the formula vol = (*D* × *d*^2^) × 0.52, where *D* is the largest diameter and *d* is the smallest diameter. All animal experiments were conducted in accordance with the Karolinska Institutet guidelines and were approved by Stockholm’s Ethical Committee of Animal Research.

### Drugs

Afatinib (BIBW2992) and R-crizotinib (PF-02341066) were purchased from SelleckChem and were stored as per the manufacturer’s recommendation.

### Whole genome sequencing

DNA was extracted from all cell lines used by using the Allprep universal kit. This DNA was quantified using NanoDrop 2000 instrument and 100 ng was subjected to WGS using library build-up with the Nextera DNA library prep, Illumina platform, and in-house developed post-read filtering (Science for Life Laboratory, Stockholm, Sweden). The resulting reads were mapped and variants called, filtering for variants in the coding regions and excluding indels, using the lab edition Partek Flow software and DNA-Seq Toolkit for Partek Flow.

### Small interfering RNA

Small interfering RNA (siRNA) sequences (smartpool) indicated in Supplementary Table 5 (Dharmacon) were used to knock down MET, WEE1, and EGFR. The Non-targeting negative control siRNA (Dharmacon) was used as Non-targeting RNA control. All siRNA were transfected using Lipofectamine 2000 (Sigma-Aldrich Chemie Gmbh, Munich, Germany) according to the manufacturer’s recommendations.

### RNA extraction

Cell line and tumor RNA extraction was performed using the product manual, using AllPrep DNA/RNA/miRNA kit (Qiagen, Hilden, Germany). RNA quantity and quality measurements were performed using Agilent Bioanalyzer 2100 instrument (Agilent Technologies, Inc., Santa Clara, CA, USA).

### Targeted sequencing using Ion AmpliSeq™

Targeted sequencing of 20,802 different transcripts was performed using the Ion AmpliSeq Transcriptome Human Gene Expression Kit for RefSeq genes (Thermo Fisher Scientific, Waltham, MA, USA). Fine-needle aspirate or core biopsy RNA from metastases and RNA from cell lines were used as input material at the Uppsala Genome Center, Uppsala University, Sweden.

BAM files were imported into the Partek Genomics Suite® 7.17.1222 software and were analyzed using their built-in RNA-sequencing workflow. Briefly, for each sample, total number of alignments, total number of reads, and percentage of reads that overlap completely, partially, or not with exonic regions were determined. Number of counts for each transcript was normalized using the reads per kilobase per million reads (RPKM) method. Comparison of mRNA abundance of candidate transcripts among samples was done using the RPKM values.

### 2D proliferation assay

For obtaining synergy plots, 800–1000 cells/well were plated overnight in a 384-well plate and DMSO as control or drugs were dispensed using a D300 digital dispenser (Hewlett-Packard, Tecan Trading AG, Switzerland). After 72 h treatment of the cells, resazurin (Sigma-Aldrich Chemie Gmbh, Munich, Germany) was added and relative fluorescence was measured using a plate reader (Tecan Spark 10 M, Tecan Trading AG, Switzerland) at 530–570 nm (excitation) and at 590–620 nm (emission). Synergy scores were calculated using Synergy Finder web application.

### MTS assay

Approximately 3000–4000 cells/well were plated overnight in 96-well flat-bottomed plates. The next day, cells were exposed to either afatinib or crizotinib (Sellekchem) alone or in combination for 72 h after which MTS solution (Promega, Madison, WI, USA) was added and absorbance at 490 nM was measured using Tecan Spark 10 M plate reader (Tecan Trading AG, Switzerland), to determine the inhibitory concentration of the drugs according to the manufacturer’s protocol. CDI was calculated as CDI = *AB*/(*A* × *B*). According to the absorbance of each group, *AB* is the ratio of the combination groups to DMSO group; *A* or *B* is the ratio of the single-agent group to the DMSO group.

### 3D MTS assay using tumor sphere growth with the hanging drop method

Approximately 10,000 cells/well were pipetted into conical well ULA plates (Corning art. 7007, Sigma-Aldrich Chemie Gmbh, Munich, Germany) in DMEM medium. To each well with 200 µl media and cells, additional medium was added to overfill the wells. Lids were attached using spacers, to allow room for the hanging drops before turning the plates. Plates were shaken at 300 r.p.m. with an amplitude of 3 mm on a lab shaker (Thermo Scientific, Waltham, MA, USA) at 37 °C in a cell incubator overnight. Plates were turned back, excess media removed, and the spheres were left to mature for 3–5 days, before being treated with single drug or a combination of drugs for 72 h. The 3D MTS solution CellTiter 3D (Promega, Madison, WI, USA) was added according to the manufacturer’s protocol. The plate was wrapped in aluminum foil and mixed at 30 r.p.m. on a laboratory rocker (Thermo Fisher Scientific, Waltham, MA, USA) for cell lysis (30 min at 37 °C). Fifty microliters of the lysate was read on a luminescence plate for ATP determination in Tecan Spark 10 M microplate reader (Tecan Trading AG, Switzerland) and drug efficacy on viability. CDI values were calculated as for the 2D viability assays.

### Immunoblotting

For western blottings for 2D and 3D cultures, cells were lysed on ice using RIPA buffer (25 mM Tris•HCl pH 7.6, 150 mM NaCl, 1% NP-40, 1% sodium deoxycholate, 0.1% SDS), 1 mM NaOV, protease and phosphatase inhibitors for 30 min, and vortexed every 10 min. Debris was removed by centrifugation and protein was measured using BCA kit as per the manufacturer’s protocol. Protein was loaded on NuPage 4–12% Bis-Tris gel (Thermo Fisher Scientific, Waltham, MA, USA) and transferred to a polyvinylidene difluoride membrane. Membranes were blocked in 5% bovine serum albumin and incubated overnight with primary antibodies against pEGFR Y845 (1:500), pEGFR Y1173(1:500), pHER2 (1:500), pHER3 Y1289(1:500), pMET Y1234/35(1:500), pAXL Y779(1:500), pERK p-44/42(1:1000), pAKT S473(1:1000), pWEE1 S642 (1:1000), and pIGF1R (1:1000). The same membranes were used for detecting EGFR (1:1000), HER2 (1:1000), HER3 (1:1000), MET (1:1000), ALK (1:1000), ERK (1:1000), AKT (1:1000), AXL (1:1000), WEE1 (1:1000), IGF1R (1:1000), TGFβ1 (1:1000), PI3KC2a (1:1000), and Actin (1:5000) after stripping with 0.4 M NaOH. Secondary conjugated anti-mouse (1:2000) or anti-rabbit (1:1000) and anti-biotin (1:2000) were used and detected by ECL reagent using Image Quant LAS 4000 (GE Healthcare Europe GmbH, Freiburg, Germany). pAXL was purchased from R&D Biosystems (Abingdon, UK) and PI3KC2a was purchased from Thermo Fisher Scientific (Waltham, MA, USA). All other primary and secondary antibodies were purchased from Cell Signaling Technologies (BioNordika, Stockholm, Sweden).

### Flow cytometry

For FACS analysis, 50,000 cells/well were plated in 12-well plates overnight and cells were treated with either a single or a combination treatment for 72 h. Cells were then trypsinized and collected. Pellets were washed once with 1× phosphate-buffered saline (PBS) and then stained for 10 min in the dark on ice with 2% Annexin V and 2% propidium iodide solution (Sigma-Aldrich Chemie Gmbh, Munich, Germany). Additional 200 µL FACS incubation buffer was added after incubation and analysis was performed using Novocyte 3000 and Novoexpress software (ACEA Biosciences, San Diego, CA, USA) to determine induction of apoptosis and necrosis.

### Scratch assay

Cells were plated to 90% confluency in 3.5 cm dishes. A wound was created using a 100 µL filter tip. Cells were exposed to either DMSO, afatinib, or crizotinib alone or in combination for 120 h. After a single 1× PBS wash, the gap-filling/wound-healing was documented using picture documentation Nikon Eclipse TS-100 microscope after 0, 6, 24, 48, and 120 h treatment. Analysis of the gap-filling/wound-healing was performed using ImageJ software.

### Transwell migration assay

Approximately 3–5 × 10^4^ cells were plated on inserts (CLS3422-48EA) with media supplemented with 2% FBS containing either DMSO, single drugs, or the combination. Seven hundred and fifty microliters of media containing 7.5% FBS was plated on the lower well (used as an attractant) and cells were allowed to migrate for 16–24 h after which they were washed in PBS, fixed in 4% formaldehyde for 5 min, followed by 20 min in methanol. Cells were finally stained using crystal violet and were imaged using Olympus Provis microscope. To measure the number of migrated cells, the crystal violet was dissolved in methanol and absorbance was measured at 540 nm using Tecan Spark 10 M plate reader instrument.

### Colony formation assay

Two hundred to 500 cells/well were plated in 6-well plates overnight. Cells were treated with either single or combination treatment for 5 days after which the media was replaced with regular media without drugs. Colonies were allowed to form for an additional 7 days, with the media being replaced every 3 days. Cells were fixed after 12 days for 20 min using 4% buffered formaldehyde. Colonies were stained with 0.05% crystal violet solution for 10 min following two washes with 1× PBS. Stained plates were scanned using Epson scanner V370. To estimate the amount of colony formation, crystal violet was dissolved in 100% methanol. In a 96-well plate, the crystal violet was diluted in 1:10 and absorbance was measured at 540 nm using Tecan Spark 10 M plate reader instrument.

### 3D invasion assay

Ten microliters of 100% Matrigel (Sigma-Aldrich Chemie Gmbh, Munich, Germany, Corning art. 356230) was used to coat the bottom of each well of an eight-chamber slide. A suspension containing 15,000 cells/mL and 4% Matrigel was added on top of the first coating. Spheres were allowed to form for 6 days after which the media was replaced with media containing DMSO, afatinib, or crizotinib alone or in combination. The drug exposure was done for 72 h. Media was removed and each well was washed twice with 1× PBS. The slides were fixed in 4% buffered formaldehyde for 15 min and thereafter washed twice with 1× PBS, permeabilized with Triton X-100 for 1 min, and blocked with 5% horse serum for 1 h. Slides were then incubated for 1.5 h with Texas Red × Phalloidin (Thermo Fisher Scientific, Waltham, MA, USA). Slides were washed thrice with 1× PBS, mounted with Fluoroshield containing DAPI (Sigma-Aldrich Chemie Gmbh, Munich, Germany) and imaged using Zeiss AxioImager M2 microscope.

### Transmission electron microscopy

Transmission electron microscopy (TEM) was performed on all cell lines using standard procedures of glutaraldehyde fixation and cell scraping prior to embedding for TEM. With a focus on the cell periphery, the presence of autophagosomes was confirmed in cells treated with afatinib (4 µM, 6 h) or crizotinib (4 µM, 6 h), a combination of both, or vehicle control (DMSO).

### Immunocytochemistry

Spheres were cultured and treated as previously described. The spheres were collected into separate tubes containing 1× cold PBS with protease inhibitor cocktail (Sigma-Aldrich Chemie Gmbh, Munich, Germany, art. 04693159001). The precipitated spheres were washed and fixed in 4% buffered formaldehyde for 1 h at RT. Formaldehyde was replaced with 70% ethanol before baking into paraffin blocks. Four-micrometer sections were cut from the paraffin-embedded blocks. Paraffin-embedded tissue sections were de-paraffinized, rehydrated, and subjected to antigen retrieval by either citrate buffer or alkaline buffer (Abcam, Cambridge, UK). They were blocked in 3% hydrogen peroxide for 10 min (Sigma-Aldrich Chemie Gmbh, Munich, Germany, art. H1009), briefly washed, and then blocked in 2.5% horse serum in Tris Buffered Saline (TBS) for 30 min before overnight incubation with primary antibody against Ki67 (1:400) or p-H2AX. All antibodies were purchased from Cell Signaling Technologies (BioNordika, Stockholm, Sweden). The following day, sections were washed, incubated overnight with biotinylated secondary antibody (Vectastain kit, art. PK-8800) followed by incubation with streptavidin peroxidase (Vectastain kit, art. SK-4100). The sections were stained with DAB (Vectastain kit art. SK-4100, Histolab Products AB, Stockholm, Sweden) for 10 min followed by counterstaining with hematoxylin (Histolab Products AB, Stockholm, Sweden, art. 01820). Slides were then mounted using Pertex (Histolab Products AB, Stockholm, Sweden, art. 00811) and images were taken using Olympus Provis microscope.

For scoring, slides were independently evaluated by two observers (I.D., R.F.M.). The intensity was given a score of 0, 1+, 2+, or 3+ (no stain, low, moderate, and strong, respectively) and an overall % of cells with positive staining was calculated for all cells per slide.

### Immunohistochemistry

IHC analysis was performed as per the manufacturer’s protocol by Cell Signaling Technologies and Dako, respectively. Briefly, paraffin-embedded tissue sections were de-paraffinized, rehydrated, and subjected to antigen retrieval by either citrate buffer, proteinase K, or EDTA. They were then blocked in 3% hydrogen peroxide for 10 min, washed, and then blocked in 2.5% horse serum for 30 min before being incubated overnight with primary antibody against EGFR (1:50), ERBB3 (1:250), or MET (1:300). EGFR antibody was purchased from Dako (M3563). All other antibodies were purchased from Cell Signaling Technologies (BioNordika, Stockholm, Sweden). The following day, sections stained with ERBB3 and MET were incubated with rabbit signal stain boost (Vectastain art. 8114, Histolab Products AB, Stockholm, Sweden). For the sections stained with EGFR, ABC staining kit (Thermo Fischer Scientific, 32052) was used. All sections were stained with DAB for 10 min and were counterstained with hematoxylin. Slides were then mounted using Pertex (Histolab Products AB, Stockholm, Sweden) and images were collected as above.

Independent evaluation of all slides was performed by three observers (I.D., R.T., and S.E.B.). In case of discrepancies between observers, a consensus was reached on further review. The intensity (negative, low (1+), moderate (2+) or strong (3+)) and proportion of ERBB3-, EGFR-, and MET-positive tumor cells were evaluated and specimens with moderate to strong staining in 20% or more of the tumor cells were regarded as high protein-expressing tumors, whereas moderate to strong staining below 20% were regarded as low protein-expressing tumors.

For IHC analysis performed using xenograft sections, three mice per group, whose median tumor volume at the start of treatment was above the median for that group, were selected. For the scoring of the mice tumor sections, four to five random fields were selected and all cells evaluated as having moderate intensity were counted as positive.

### Statistical analysis

All statistical analyses were carried out using GraphPad Prism v.7.0 (GraphPad Software, La Jolla, CA, USA). Two-tailed Student’s *t*-test was used to compare the difference between groups. For animal experiments, Mann–Whitney test was used to calculate the differences between groups, unless otherwise stated.

## Supplementary information

Supplementary figures

Supplementary tables
