# Supplementary material for: Combining ERBB family and MET inhibitors is an effective therapeutic strategy in cutaneous malignant melanoma independent of BRAF/NRAS mutation status
Source: Cell Death Dis. 2019 Sep 10;10(9):663. doi: 10.1038/s41419-019-1875-8 (PMC6737096; doi:10.1038/s41419-019-1875-8)
Supplement: Supplementary file 1 — Supplementary figures [file 41419_2019_1875_MOESM1_ESM.pdf]

## Supplementary information captions

**Supplementary Figure S1:EGFR protein expression in CMM.**

Images showing membranous and cytoplasmic protein expression of EGFR in CMM. Please also see Supplementary table 1.

**Supplementary Figure S2: Crizotinib downregulates AKT expression**

Western blot showing that short treatment (3h) with crizotinib is sufficient to downregulate pAKT.

**Supplementary Figure S3: Synergy scores for A. A375 (BRAFV600E), B. A375VR4, C.SkMel2, D. ESTDAB105.**

a-d A375, A375VR4, SkMEl2 and ESTDAB105 treated with the drug combination shows synergistic effects when tested with the above drug dose range. Three independent experiments have been performed.

**Supplementary Figure S4: 3D spheroids cultured by hanging drop.**

Examples of 3D spheroids using a. A375 b.A375VR4c. SkMel28 d. ESTDAB037 and e. ESTDAB105 after 72h of culture (left panel= 4X, right panel= 10X)

**Supplementary Figure S5: Combination of afatinib and crizotinib in 2D and 3D cell proliferation assay.**

a. Effects of combination therapy on CMM cells measured by 2D proliferation assay. Samples are expressed as mean± SD. Three independent experiments have been performed. *p<0.05, **p<0.01,***p<0.001,****p<0.0001 by two tailed student's t test. b. Effects of combination therapy on 3D spheroids measured by 3D proliferation assay. *p<0.05, **p<0.01, ***p<0.001,****p<0.0001 by two tailed student's t test.

**Supplementary Figure S6: p-H2AX is induced upon treatment with single or combination treatments.**

ICC done on spheroids showed further induction of DNA damage marker H2AX for 3 out of the 4 cell lines tested. The experiment was repeated twice.

**Supplementary Figure S7: Autophagosomes are induced upon treatment with single or combination drug (s).**

Transmission Electron Microscopy (TEM) pictures showing that autophagosomes (marked with arrow heads) were induced in all cell lines upon treatment for 6h with 4µM of either single drug or the combination.

**Suppplementary Figure S8: CMM cells show decrease in cell migration when treated with combination therapy.**

(a).Melanoma cells show a decrease in wound healing potential after combination treatment. Cells were followed for either upto 48h or 120h depending on the migration potential of the cell line. Wound gap was measured when wound was healed for DMSO control. (b). Quantitaive comparison of wound gap remaining after 48h or 120h compared to wound gap on day 0 *$p<0.05$, **$p<0.01$, ***$p<0.001$, ****$p<0.0001$ by two tailed student's t test. Samples are expressed as mean± SD. Three independent experiments have been performed.
**$p<0.01$, ***$p<0.001$, ****$p<0.0001$

**Supplementary Figure S9: Median volume of xenograft tumors.**

(a). Tumor volume fold change over time. (b). Median volume of xenograft tumors before start of treatment. (c). After end of treatment. (d). Table showing the median volumes. E. Tumor weight at the end of treatment. All samples are expressed as mean ± SEM. Differences between animal groups were calculated by two tailed student's t test *$p<0.05$, **$p<0.01$, ***$p<0.001$, ****$p<0.0001$

**Supplementary Figure S10: Genomic mutations mapped using mutation mapper for four out of the five cell lines investigated more extensively in the study**

**Supplementary Figure S11: Western blot showing targets of afatinib and crizotinib across 5 cell lines.**

(a). Canonical target of afatinib HER2 and one of the predicted secondary targets procured from the network analysis. (b). Phosphorylated form of HER2 after stimulation with EGF for 10 minutes. Two independent experiments have been performed.

**Supplementary Figure S12: Silencing of MET alone does not sensitize CMM cells to drug treatment.**

Transfection with sicontrol or siMET in (a). A375 and (b). A375VR4 followed by treatment with afatinib (2µM or 3µM) for an additional 24h did not cause any further sensitization when compared to afatinib treatment alone. *$p<0.05$, **$p<0.01$,***$p<0.001$,****$p<0.0001$ by two tailed student's t test. All samples are expressed as mean ± SD. Three independent experiments were performed.

**Supplementary Figure S13: Knockdown of Wee1 induces DNA damage in CMM cell lines.**

Western blot showing induction of p-H2AX signal, concomitant with decrease in pAKT signal upon knockdown of Wee1.

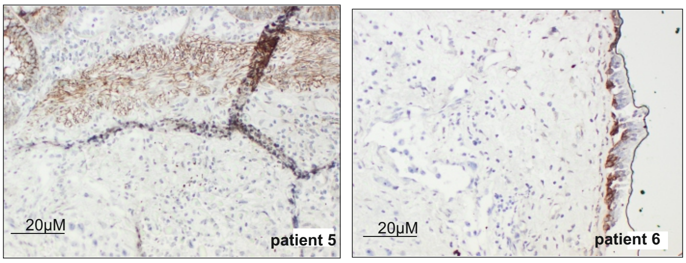


Supplementary Figure S1

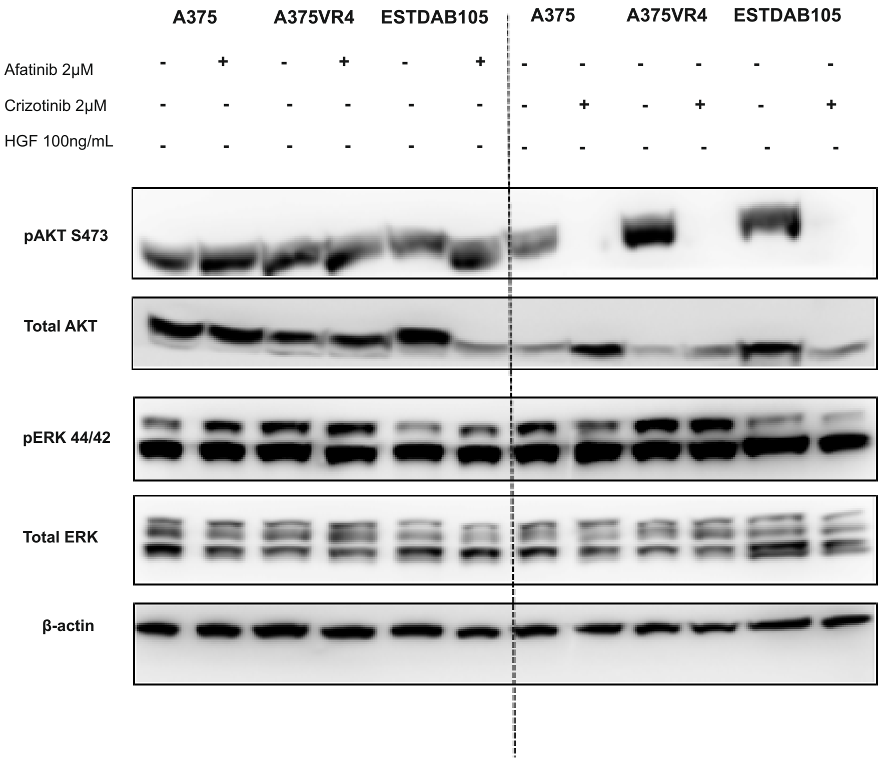


Supplementary Figure S2

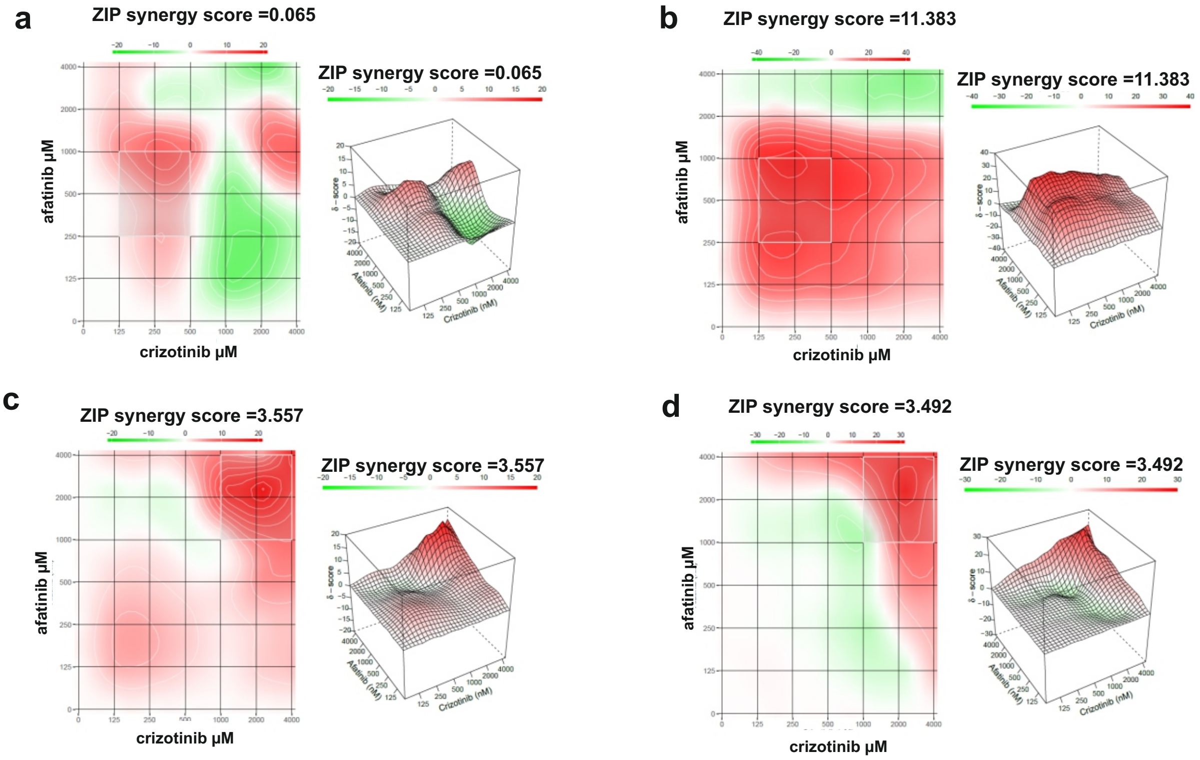


Supplementary Figure S3

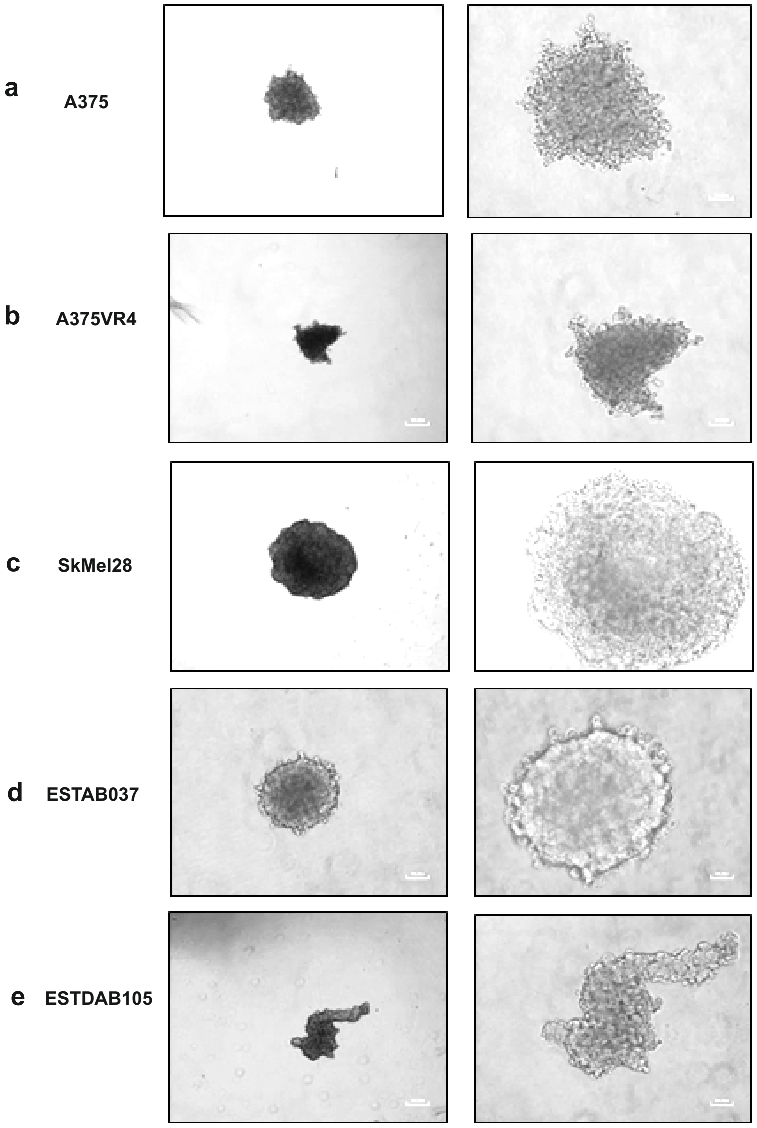


Supplementary Figure S4

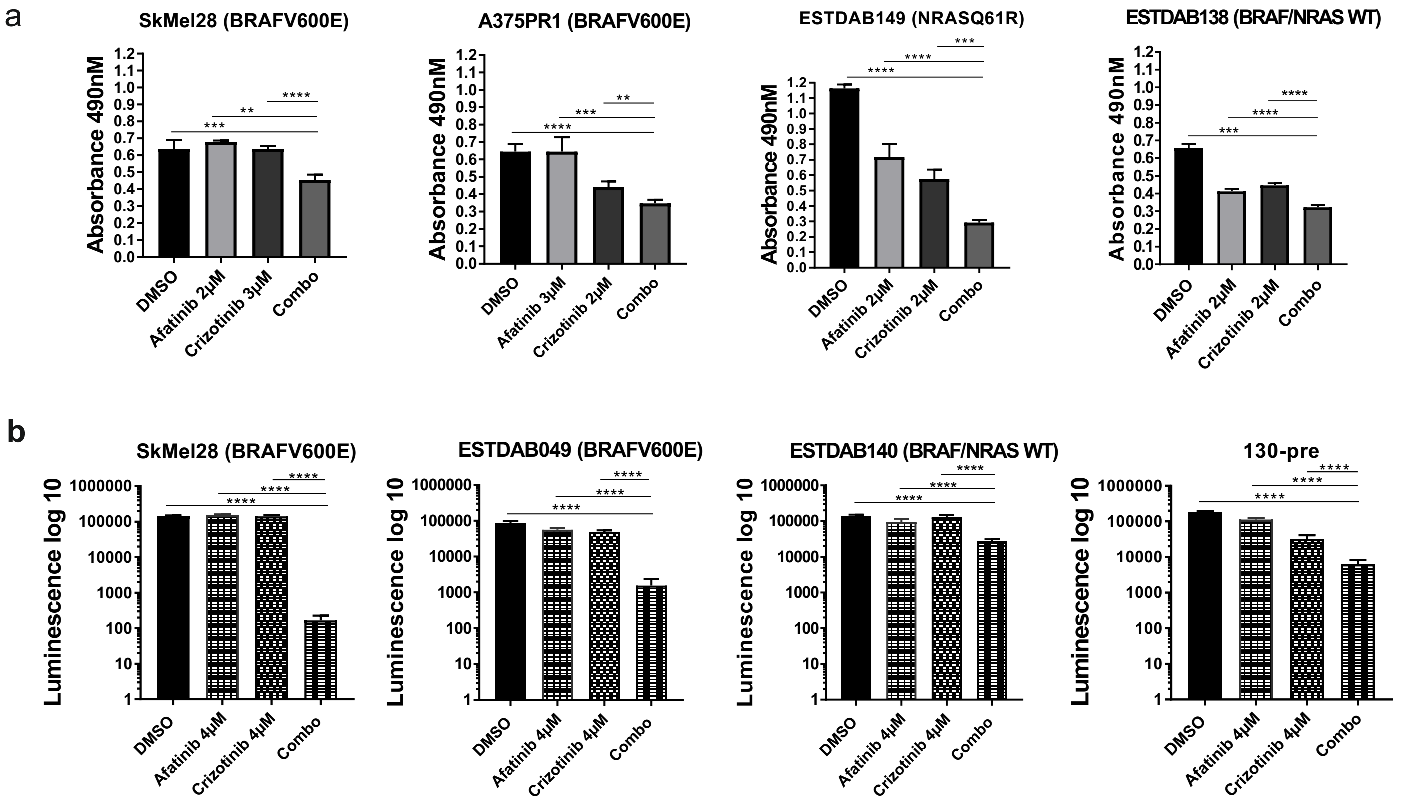


Supplementary Figure S5

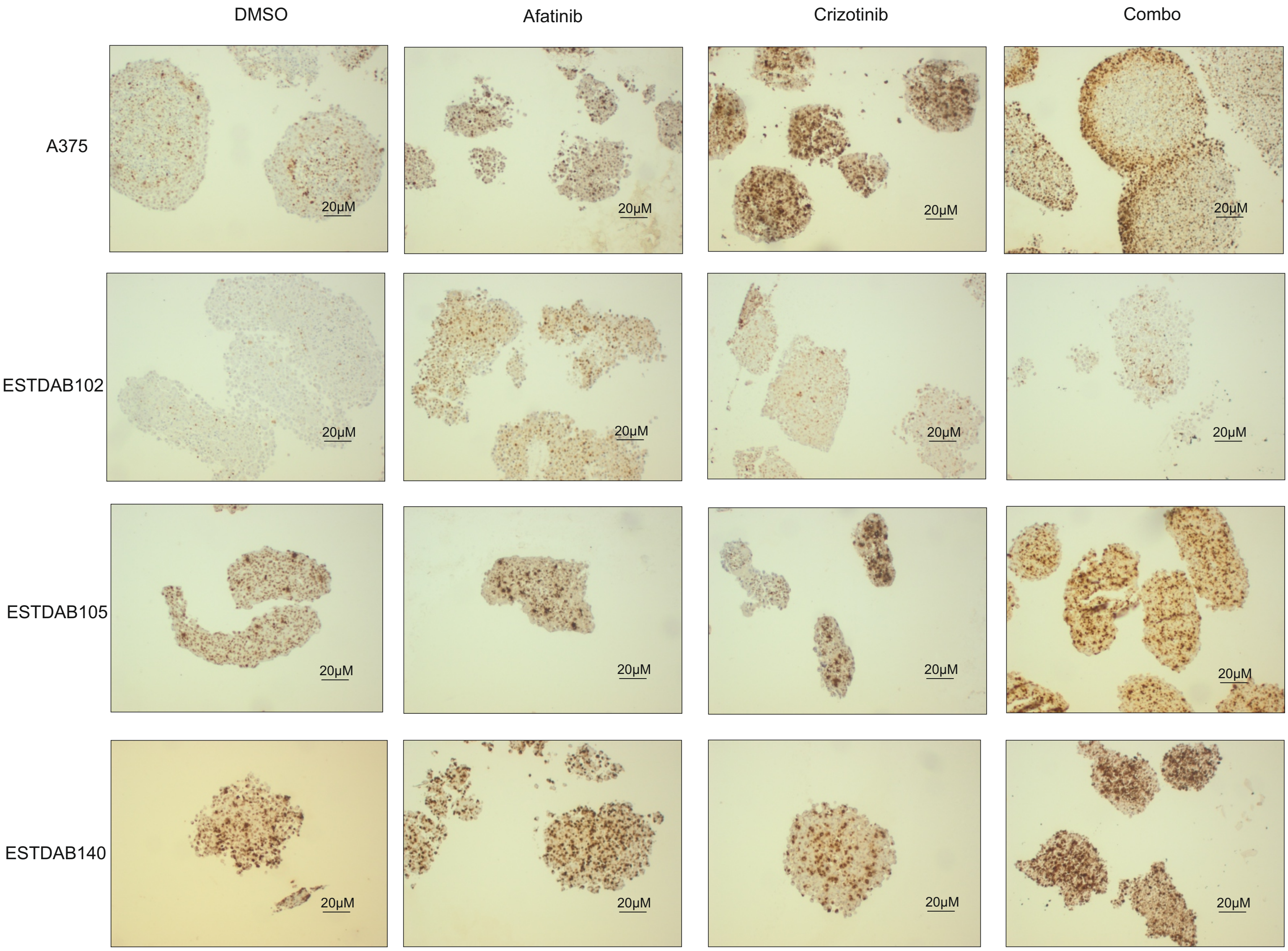


Supplementary Figure S6

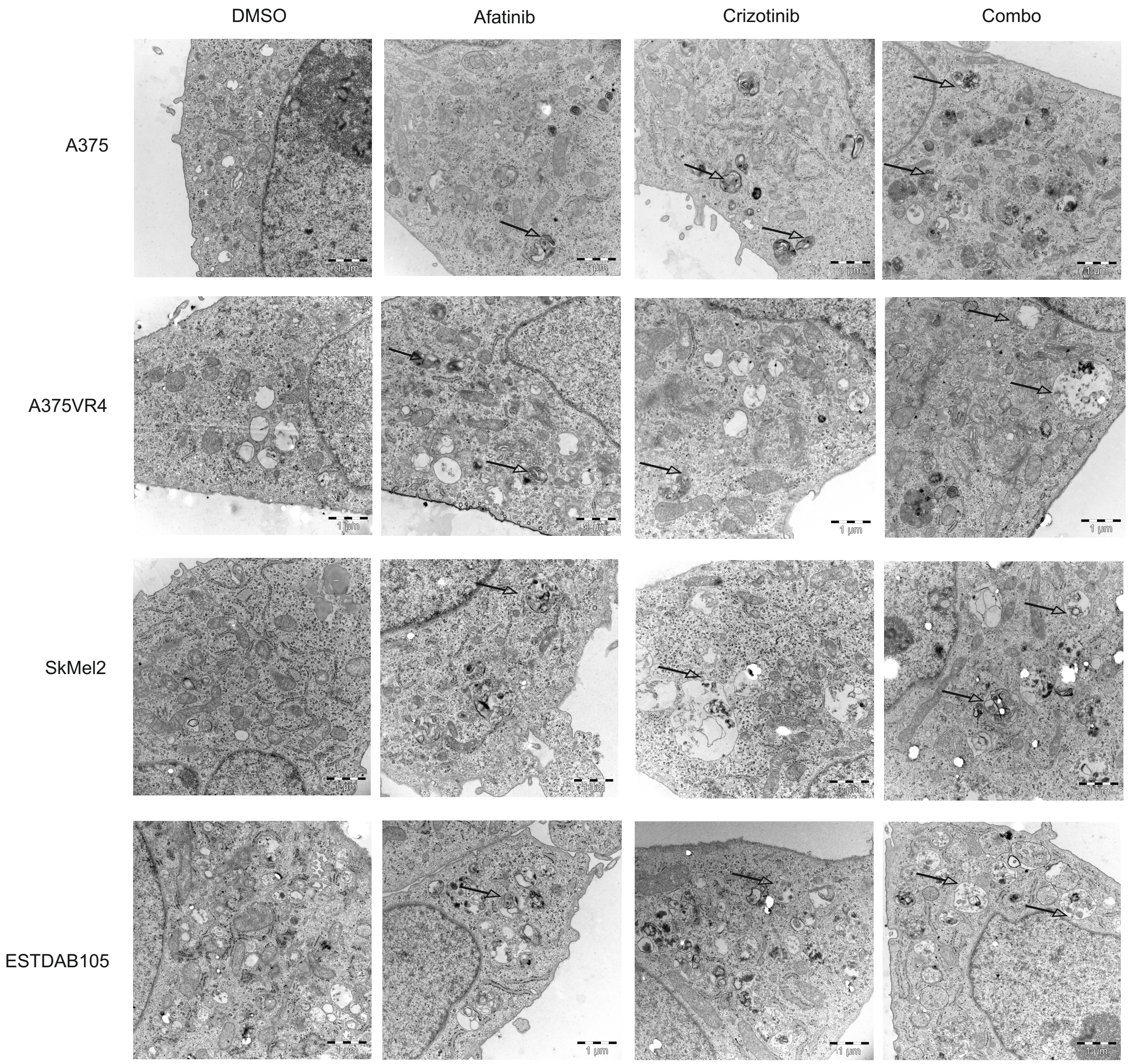


Supplementary Figure S7

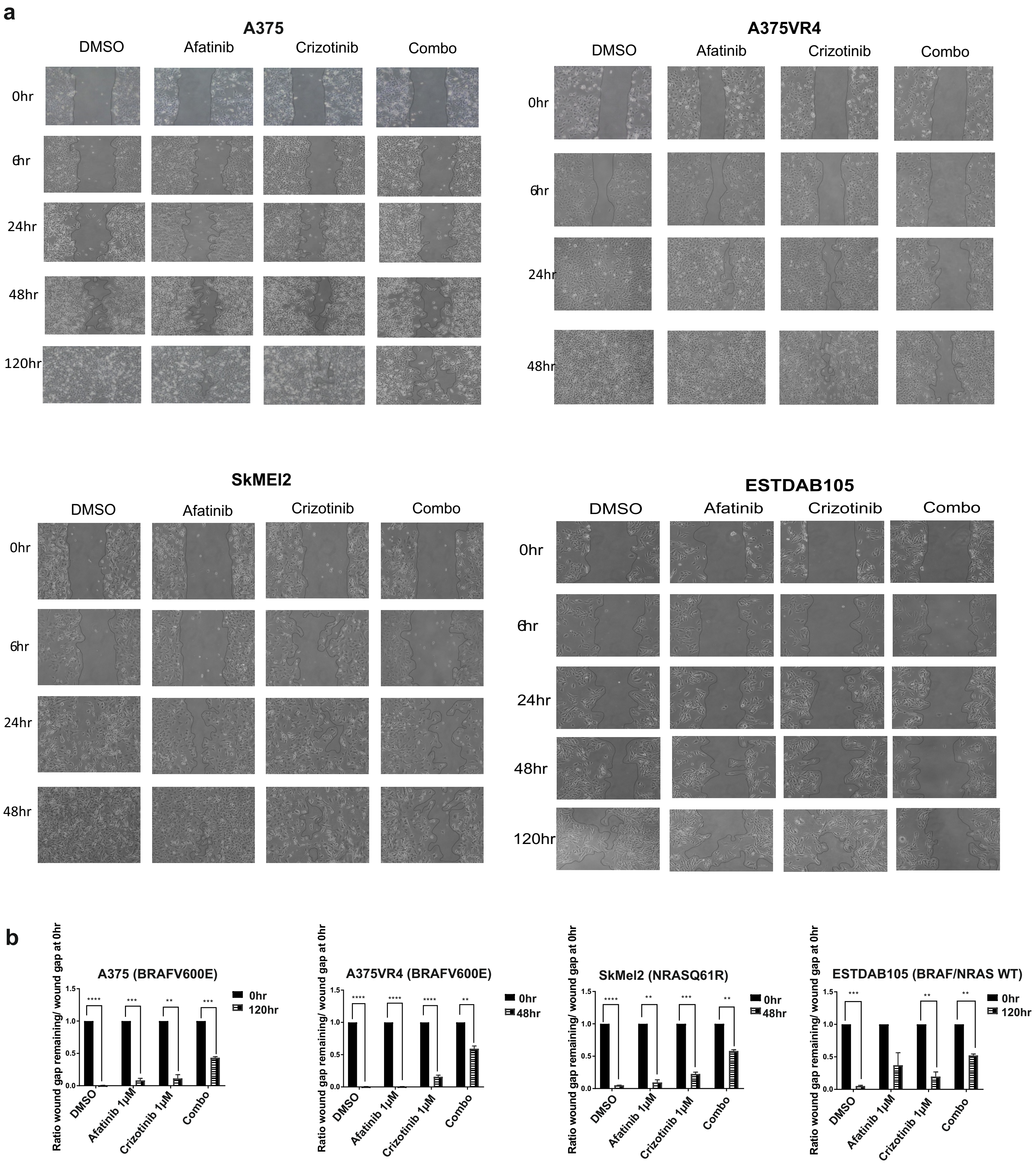


Supplementary Figure S8

a

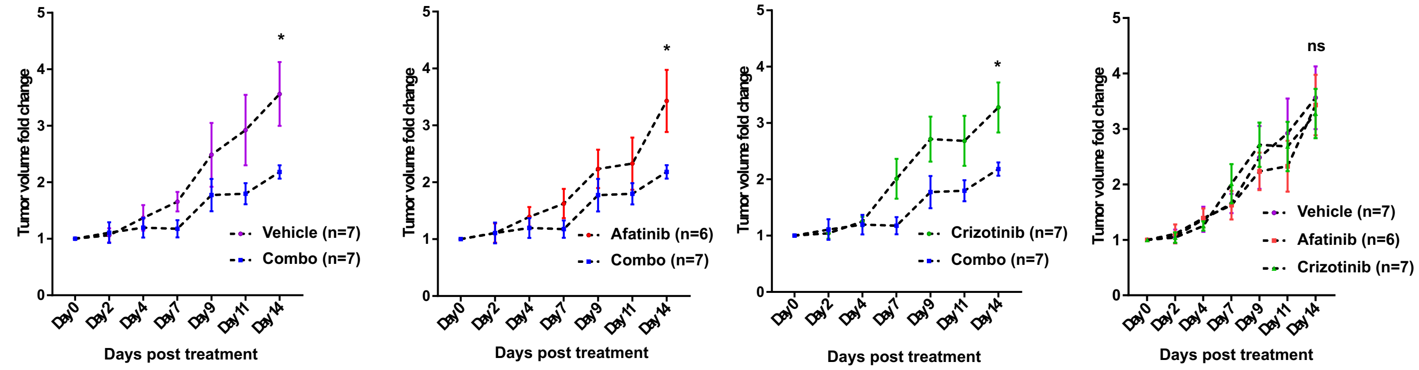


b

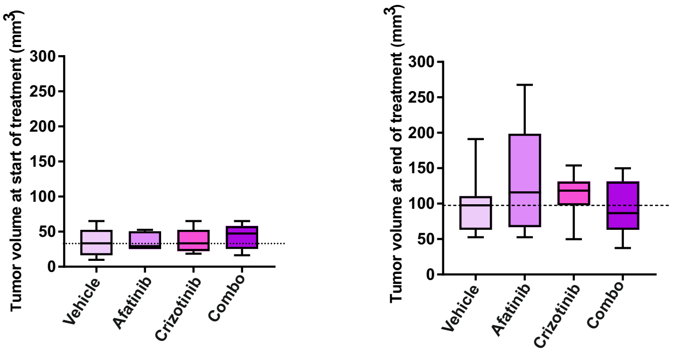


d

| | Vehicle | Afatinib | Crizotinib | Combo |
|---|---|---|---|---|
| **Median tumor volume day =0** | 33 $mm^3$ | 41 $mm^3$ | 33 $mm^3$ | 48 $mm^3$ |
| **Median tumor volume day =14** | 91 $mm^3$ | 116 $mm^3$ | 118 $mm^3$ | 86 $mm^3$ |

e

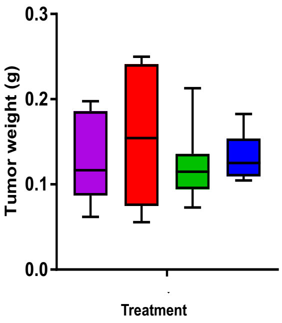


Supplementary Figure S9

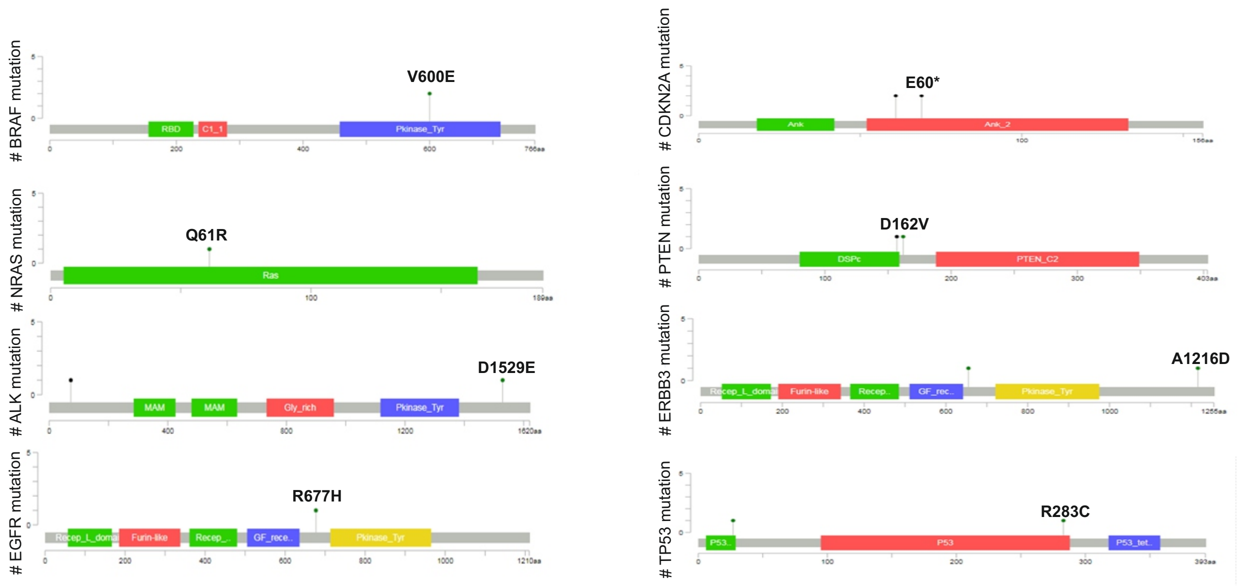


Supplementary Figure S10

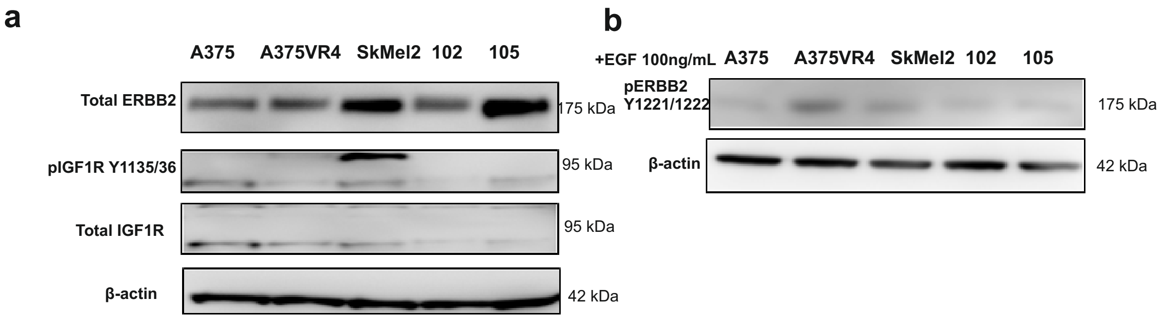


Supplementary Figure S11

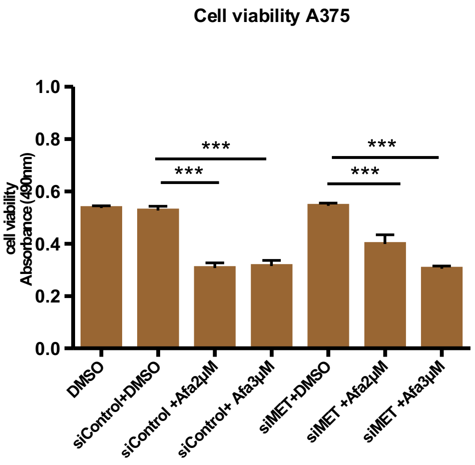


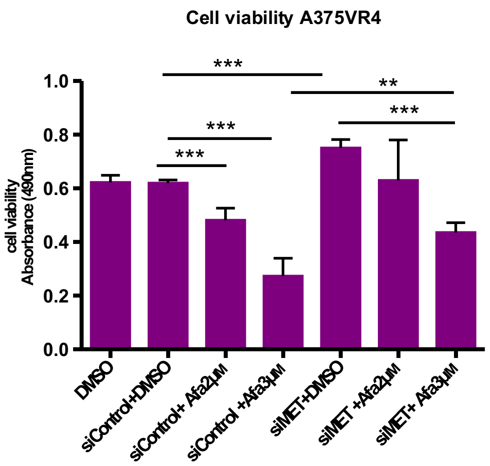


Supplementary Figure S12

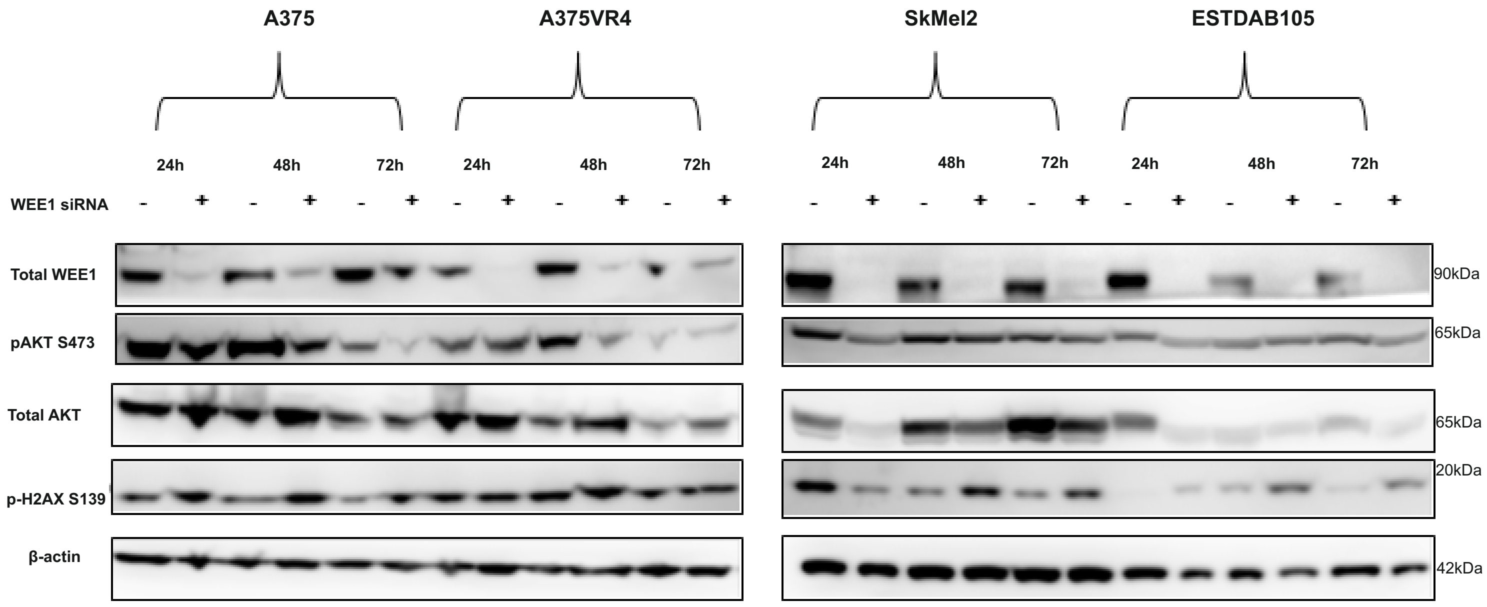


Supplementary Figure S13
