# Supplementary material for: Combining ERBB family and MET inhibitors is an effective therapeutic strategy in cutaneous malignant melanoma independent of BRAF/NRAS mutation status
Source: Cell Death Dis. 2019 Sep 10;10(9):663. doi: 10.1038/s41419-019-1875-8 (PMC6737096; doi:10.1038/s41419-019-1875-8)
Supplement: Supplementary file 2 — Supplementary tables [file 41419_2019_1875_MOESM2_ESM.pdf]

Supplementary Table 1. Summary of EGFR, MET and ERBB3 scoring in CMM tumors using IHC

protein	< 20% 2+ and/or 3+(%)	>20% 2+ and/or 3+(%)
ERBB3	8	92
MET	57	43
EGFR	86	14

Supplementary Table 2. Baseline mRNA expression status of selected RTKs in a. CMM patients **b,c.** Melanoma cell lines
*p value= difference between R and NR

a

No. of patients	Treatment response	Median and range of mRNA expression of canonical targets (RTKs) of afatinib and crizotinib							
		EGFR	p-value	ERBB2	p-value	ERBB3	p-value	MET	p-value
6	Responder (R)	34.13 (0-92.70)	0.07	14.34 (9.07-39.34)	0.37	851.16 (350.94-3701.92)	0.24	116.89 (75.50-484.96)	0.39
7	Non-responder (NR)	147.00 (2.24-377.17)		31.39 (10.94-64.07)		810.90 (8.42-2349.43)		73.32 (33.68-687.74)	

b

Genes	Relative mRNA expression				
	A375	A375VR4	SkMel2	ESTDAB102	ESTDAB105
EGFR	27.2	670.2	34.2	336.9	294.8
ERBB2	60.5	37.7	24.9	44.4	83.9
ERBB3	2384.2	343.4	3150.8	995.7	2326.9
MET	225.8	559.7	297.4	613.1	156.8
AXL	4.6	895.6	349.6	54.0	61.2
ALK	7.7	4.3	0.0	0.6	3.1
ROS1	0.0	4.8	0.6	0.0	0.0
MAPK1 (ERK2)	1185.7	405.0	332.2	628.8	762.0
MAPK3 (ERK1)	237.1	244.0	214.3	397.3	227.2
AKT1	291.3	229.0	378.2	414.1	303.0
AKT2	765.0	849.9	523.2	550.7	482.2
AKT3	1314.5	1302.7	886.5	858.2	2653.8

c

Genes	Relative mRNA expression								
	A375PR1	ESTDAB037	ESTDAB049	ESTDAB140	KADA	ANRU	121-PRE	SKMEL24	SKMEL28
EGFR	101.1	5192.4	6.5	3713.8	296.9	0.6	982.8	485.8	68.0
ERBB2	40.0	34.5	41.6	41.6	64.6	65.8	76.4	190.9	40.3
ERBB3	1847.5	0.0	530.3	2.5	132.9	694.4	42.5	1200.7	603.4
MET	531.4	167.0	1965.5	2582.1	880.7	1182.3	566.0	204.4	191.0
AXL	371.3	23.0	4.1	494.0	749.0	61.7	3.4	917.5	3.7
ALK	17.5	0,0	0,8	0,0	0,0	0,6	0,0	4,3	3,1
ROS1	0.0	8.4	0.0	47.9	94.4	0.0	0.0	0.0	0.0
MAPK1 (ERK2)	977.1	619.0	641.3	276.0	509.3	883.6	851.9	693.3	2092.8
MAPK3 (ERK1)	277.4	315.1	234.8	135.3	334.8	411.9	146.6	318.8	367.5
AKT1	302.4	286.1	203.7	120.5	162.5	97.0	301.6	492.1	257.6
AKT2	783.4	530.9	544.2	389.4	617.3	489.7	539.1	899.0	634.8
AKT3	1933.9	1676.2	2156.4	1224.9	1040.9	1425.3	1138.4	913.2	1096.4

Supplementary Table 3. IC50 and IC30 values of afatinib and crizotinib in melanoma cell lines. *ND= Not determined

Cell line	IC50 Afatinib	IC50 Crizotinib
A375 (*BRAF*V600E)	2.10μM	2.30μM
A375VR4(*BRAF*V600E)	3.30μM	2.94μM
SkMel24(*BRAF*V600E)	2.80μM	4.79μM
SkMel28(*BRAF*V600E)	4.98μM	ND*
SkMel2(*NRAS*Q61R)	2.49μM	0.60μM
ESTDAB102(*NRAS*Q61R)	3.90μM	8.41μM
ESTDAB105(WT)	2.45μM	3.30μM
ESTDAB138(WT)	1.80μM	2.30μM

Cell line	IC30 Afatinib	IC30 Crizotinib
A375 (*BRAF*V600E)	1.49μM	2.07μM
A375VR4(*BRAF*V600E)	2.68μM	2.19μM
SkMel24(*BRAF*V600E)	2.15μM	3.2μM
SkMel28(*BRAF*V600E)	3.53μM	ND*
SkMel2(*NRAS*Q61R)	2.10μM	0.35μM
ESTDAB102(*NRAS*Q61R)	2.8μM	6.12μM
ESTDAB105(WT)	1.47μM	2.11μM
ESTDAB138(WT)	1.52μM	1.51μM

Supplementary Table 4. Whole genome sequencing results showing the medium and high annotation impact variants of selected genes

Cell line	Chr	Position	ID	Refs	Alts	Annotation	Gene name	HGVS.c	HGVS.p	AD	Genotype
**ESTDAB102**	1	115256529	rs11554290	T	C,<NON_REF>	missense_variant	NRAS	c.182A>G	p.Gln61Arg	10,62,0	TC
**A375**	2	29416572	rs1670283	T	C	missense_variant	ALK	c.4381A>G	p.Ile1461Val	0,27	CC
**A375VR4**	2	29416572	rs1670283	T	C,<NON_REF>	missense_variant	ALK	c.4381A>G	p.Ile1461Val	0,34,0	CC
**ESTDAB102**	2	29444095	rs1569156	C	T,<NON_REF>	stop_gained	ALK	c.218G>A	p.Trp73*	0,47,0	TT
**ESTDAB102**	2	29509701		C	A,<NON_REF>	splice_acceptor_variant&intron_variant	ALK	n.373-1G>T		38,2,0	CC
**ESTDAB102**	2	29416572	rs1670283	T	C,<NON_REF>	missense_variant	ALK	c.4381A>G	p.Ile1461Val	0,41,0	CC
**ESTDAB105**	2	29416366	rs1881421	G	C,<NON_REF>	missense_variant	ALK	c.4587C>G	p.Asp1529Glu	24,11,0	GC
**ESTDAB105**	2	29416572	rs1670283	T	C,<NON_REF>	missense_variant	ALK	c.4381A>G	p.Ile1461Val	0,30,0	CC
**A375**	7	140453136	rs113488022	A	T	missense_variant	BRAF	c.1799T>A	p.Val600Glu	0,33	TT
**A375VR4**	7	140453136	rs113488022	A	T,<NON_REF>	missense_variant	BRAF	c.1799T>A	p.Val600Glu	0,30,0	TT
**A375**	7	55229255	rs2227983	G	A	missense_variant	EGFR	c.1562G>A	p.Arg521Lys	12,23	GA
**A375VR4**	7	55229255	rs2227983	G	A,<NON_REF>	missense_variant	EGFR	c.1562G>A	p.Arg521Lys	13,32,0	GA
**ESTDAB102**	7	55229255	rs2227983	G	A,<NON_REF>	missense_variant	EGFR	c.1562G>A	p.Arg521Lys	13,27,0	GA
**ESTDAB105**	7	55229255	rs2227983	G	A,<NON_REF>	missense_variant	EGFR	c.1562G>A	p.Arg521Lys	12,23,0	GA
**A375**	9	21971153	rs121913383	C	A	stop_gained	CDKN2A	c.205G>T	p.Glu69*	1,1	AA
**A375**	9	21971177	rs121913382	C	A	stop_gained	CDKN2A	c.181G>T	p.Glu61*	0,8	AA
**A375VR4**	9	21971153	rs121913383	C	A,<NON_REF>	stop_gained	CDKN2A	c.205G>T	p.Glu69*	0,14,0	AA
**A375VR4**	9	21971177	rs121913382	C	A,<NON_REF>	stop_gained	CDKN2A	c.181G>T	p.Glu61*	0,15,0	AA
**ESTDAB102**	10	89720649		A	T,<NON_REF>	splice_acceptor_variant&intron_variant	PTEN	c.802-2A>T		24,2,0	AA
**ESTDAB105**	10	89693001		A	T,<NON_REF>	missense_variant	PTEN	c.485A>T	p.Asp162Val	1,23,0	TT
**ESTDAB105**	10	89692985	rs121909220	G	T,<NON_REF>	stop_gained	PTEN	c.469G>T	p.Glu157*	0,27,0	TT
**ESTDAB102**	15	99496340	rs12148482	G	T,<NON_REF>	splice_donor_variant&intron variant	IGF1R	n.316+1G>T		0,30,0	TT
**ESTDAB105**	15	99467831	rs56400113	C	T,<NON_REF>	sequence_feature	IGF1R	c.2700C>T		10,40,0	CT
**A375**	17	37884037	rs1058808	C	G	missense_variant	ERBB2	c.3508C>G	p.Pro1170Ala	12,13	CG
**A375VR4**	17	37884037	rs1058808	C	G,<NON_REF>	missense_variant	ERBB2	c.3508C>G	p.Pro1170Ala	12,13	CG
**ESTDAB105**	17	37879588	rs1136201	A	G,<NON_REF>	missense_variant	ERBB2	c.1963A>G	p.Ile655Val	18,10,0	AG
**ESTDAB105**	17	37884037	rs1058808	C	G,<NON_REF>	missense_variant	ERBB2	c.3508C>G	p.Pro1170Ala	0,33,0	GG
**ESTDAB105**	17	37884176	rs55943169	C	A,<NON_REF>	missense_variant	ERBB2	c.3647C>A	p.Ala1216Asp	19,9,0	CA
**A375**	17	7579472	rs1042522	G	C	missense_variant	TP53	c.215C>G	p.Pro72Arg	20,12	GC
**A375VR4**	17	7579472	rs1042522	G	C,<NON_REF>	missense_variant	TP53	c.215C>G	p.Pro72Arg	24,16,0	GC
**ESTDAB102**	17	7579472	rs1042522	G	C,<NON_REF>	missense_variant	TP53	c.215C>G	p.Pro72Arg	0,25,0	CC
**ESTDAB105**	17	7577091	rs149633775	G	A,<NON_REF>	missense_variant	TP53	c.847C>T	p.Arg283Cys	14,26,0	GA
**ESTDAB105**	17	7579472	rs1042522	G	C,<NON_REF>	missense_variant	TP53	c.215C>G	p.Pro72Arg	0,39,0	CC
**ESTDAB105**	17	7579717		G	A,<NON_REF>	missense_variant	TP53	c.79C>T	p.Pro27Ser	13,22,0	GA
**A375**	19	41743861	rs7249222	A	G	missense_variant	AXL	c.796A>G	p.Asn266Asp	0,29	GG
**A375VR4**	19	41743861	rs7249222	A	G,<NON_REF>	missense_variant	AXL	c.796A>G	p.Asn266Asp	0,35,0	GG
**ESTDAB102**	19	41743861	rs7249222	A	G,<NON_REF>	missense_variant	AXL	c.796A>G	p.Asn266Asp	0,21,0	GG
**ESTDAB105**	19	41743861	rs7249222	A	G,<NON_REF>	missense_variant	AXL	c.796A>G	p.Asn266Asp	0,32,0	GG

Supplementary Table 5. Table showing siRNA sequences used to silence MET, WEE1 and EGFR

Sequence number	Target Sequence
MET siRNA #1	5'- GAACUGGUGUCCCGGAUAU-3'
MET siRNA #2	5'- GAACAGCGAGCUAAAUAUA-3'
MET siRNA #3	5'- GAGCCAGCCUGAAUGAUGA-3'
MET siRNA #4	5'- GUAAGUGCCCGAAGUGUAA-3'
WEE1 siRNA #1	5'-AAUAGAACAUCUCGACUUA-3'
WEE1 siRNA #2	5'-AAUAUGAAGUCCCGGUAUA-3'
WEE1 siRNA #3	5'-GAUCAUAUGCUUAUACAGA-3'
WEE1 siRNA #4	5'-CGACAGACUCCUCAAGUGA-3'
EGFR siRNA #1	5'-CAAAGUGUGUAACGGAAUA-3'
EGFR siRNA #2	5'-CCAUAAAUGCUACGAAUAU-3'
EGFR siRNA #3	5'-GUAACAAGCUCACGCAGUU-3'
EGFR siRNA #4	5'-CAGAGGAUGUUCAAUAACU-3'
